# First-Principles Studies of the Electronic and Optical Properties of Zinc Titanium Nitride: The Role of Cation Disorder

**DOI:** 10.1021/acs.chemmater.3c02696

**Published:** 2024-03-25

**Authors:** Sijia Ke, John S. Mangum, Andriy Zakutayev, Ann L. Greenaway, Jeffrey B. Neaton

**Affiliations:** † Department of Materials Science and Engineering, University of California at Berkeley, Berkeley, California 94720, United States; ‡ Chemical Sciences Division, Lawrence Berkeley National Laboratory, Berkeley, California 94720, United States; § Materials, Chemistry, and Computational Science Directorate, National Renewable Energy Laboratory, Golden, Colorado 80401, United States; ∥ Department of Physics, University of California at Berkeley, Berkeley, California 94720, United States; ⊥ Materials Sciences Division, Lawrence Berkeley National Laboratory, Berkeley, California 94720, United States; # Kavli Energy NanoSciences Institute at Berkeley, Berkeley, California 94720, United States

## Abstract

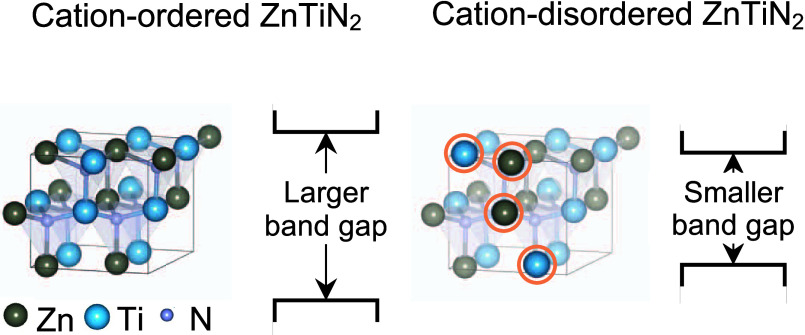

Cation disorder is an established feature of heterovalent ternary nitrides, a promising class of semiconductor materials. A recently synthesized wurtzite-family ternary nitride, ZnTiN_2_, shows potential for durable photoelectrochemical applications with a measured optical absorption onset of 2 eV, which is 1.4 eV lower than previously predicted, a large difference attributed to cation disorder. Here, we use first-principles calculations based on density functional theory to establish the role of cation disorder in the electronic and optical properties of ZnTiN_2_. We compute antisite defect arrangement formation energies for one hundred 128-atom supercells and analyze their trends and their effect on electronic structures, rationalizing experimental results. We demonstrate that charge imbalance created by antisite defects in Ti and N local environments, respectively, broadens the conduction and valence bands near the band edges, reducing the band gap relative to the cation-ordered limit, a general mechanism relevant to other multivalent ternary nitrides. Charge-imbalanced antisite defect arrangements that lead to N-centered tetrahedral motifs fully coordinated by Zn are the most energetically costly and introduce localized in-gap states; cation arrangements that better preserve local charge balance have smaller formation energies and have less impact on the electronic structure. Our work provides insights into the nature of cation disorder in the newly synthesized semiconductor ZnTiN_2_, with implications for its performance in energy applications, and provides a baseline for the future study of controlling cation order in ZnTiN_2_ and other ternary nitrides.

## 1 Introduction

For several decades, nitride semiconductors have been central to optoelectronics and energy applications, including but not limited to light-emitting diodes and laser diodes,^1^ electrochemical energy storage,^2^ photovoltaic devices,^3^ and more.^4,5^ Although binary nitrides comprise the vast majority of semiconductors used for these applications, relatively fewer ternary nitrides have been explored or are in use.^5−7^ Recent computational searches^6,8−10^ have identified some new promising functional ternary nitrides, but few^10−18^ have been synthesized as yet due to challenges associated with the relative inertness of nitrogen.

Recently, the previously predicted^6,8^ ternary nitride ZnTiN_2_ was synthesized for the first time.^12,19^ As synthesized, ZnTiN_2_ is semiconducting with a measured band gap of about 2 eV, has a wurtzite-derived structure that can be well integrated with high-performance semiconductors (like GaN), and may form self-passivating layers under electrochemical operation conditions, making it promising for solar fuels and electrochemical applications.^12^

ZnTiN_2_ is part of a class of wurtzite-derived heterovalent ternary nitride semiconductors that feature a significant concentration of cation antisite defects in thin films, the so-called cation disorder, which arises during synthesis due to low antisite defect formation energies^20−22^ and limited cation kinetics under synthesis conditions.^10,23^ Cation disorder is a central feature of these ternary nitride thin films, and it affects the electronic, optoelectronic, and transport properties. While X-ray diffraction measurements can detect cation disorder,^20^ experiments have been limited in probing its details,^10,24,25^ and the local atomic-scale distribution and concentration of cations remain an open question. For other heterovalent ternary nitrides, such as ZnGeN_2_^20,22,26−31^ and ZnSnN_2_,^21,26,27,32−35^ first-principles density functional theory (DFT) calculations of large supercells containing antisite defects have shown that the band gap and band structure are relatively insensitive to cation disorder as long as the sum of the nominal valence of the cations coordinating the nitrogen anion cancels its nominal valence (−3), enforcing local charge neutrality.^26^ Lany et al.^32^ used effective Hamiltonians based on DFT to calculate energetics of antisite defects and performed Monte Carlo simulations to generate cation-disordered structures of ZnSnN_2_ at different temperatures; ref (32) found that the DFT band gap of cation-disordered ZnSnN_2_ decreases gradually, by about 0.6 eV for up to 45% charge-imbalanced motifs, with an increasing number of charge-imbalanced motifs becoming more stable with rising temperature.^32^ Using cluster-expanded Hamiltonians, Cordell et al.^30,31^ found that ZnGeN_2_ exhibits a first-order phase transition in cation disorder density, with the system predicted to go from more than 90% to around 50% local charge-neutral motifs (low-to-high cation disorder) above an effective temperature of 2500 K, resulting in a large 2–3.5 eV DFT band gap reduction in the high-cation disorder regime.

Despite prior reports of cation disorder-reduced band gaps in zinc ternary nitrides,^20,26,30−32^ the physical origin of this behavior remains unclear and controversial.^5^ Some reports suggest that a low number of antisite defects introduce localized midgap states that become increasingly dispersive with growing defect concentration.^28^ Other studies report that cation disorder broadens the tail of the band edge.^32^ Moreover, the effect of cation disorder can depend on the chemistry of the specific ternary nitride. Compared to ZnSnN_2_, ZnGeN_2_ is reported to experience a larger DFT band gap reduction by about 1 eV at similar concentrations of antisite defects.^26,31,32^ For ZnTiN_2_, disorder associated with the Ti^4+^ cations, with their unoccupied 3d shells, would be likely to alter the electronic structure and band gap differently than that of Ge and Sn in their associated zinc ternary nitrides.^12^

In this work, we use state-of-the-art first-principles methods based on DFT on a number of large supercells to compute the energetics of cation disorder and its effect on the electronic and optoelectronic properties of ZnTiN_2_. Cation disorder via introduction of antisite defects disrupts the local balance of Ti and Zn cations and leads to charge-imbalanced N-centered tetrahedral motifs, as well as clusters of these tetrahedra that contain a majority of the same cation species, with larger charge imbalance and greater formation energies. We elucidate the physical mechanism behind the cation disorder-induced band gap reduction: the chemically heterogeneous local environments lead to broadening of valence and conduction bands near the band edges relative to the ordered crystal, reducing the band gap. Positive and negative local charge imbalances are found to have different effects on the electronic structure. Additionally, Zn cation clustering is computed to have a larger formation energy relative to other local cation arrangements. As an example of extreme Zn cation clustering, the local arrangement where four Zn fully coordinate one N introduces localized states above the valence band edge of the cation-ordered system at a higher energy and leads to a greater reduction in the band gap. While cation disorder without extreme Zn clustering does not lead to localized in-gap states, conduction band edge states are dominated by the Ti d orbital character in the case of high positive charge imbalance, decreasing electron carrier mobility relative to the cation-ordered limit. The optical absorption spectra of high cation-disordered supercells, i.e., where around or more than 50% of the N-centered tetrahedral motifs are charge-imbalanced, are computed with linear-response time-dependent DFT using hybrid functionals, and good agreement is obtained in compared experiments. Our calculations agree best with measurements for high cation-disordered supercells, providing insights into the atomic nature of the cation disorder in samples synthesized thus far. Our understanding of the relation between the cation disorder and the band gap can be extended to other heterovalent ternary nitrides, shedding light on the nature of cation disorder and the future development of cation ordering manipulation in a broader class of nitride semiconductors.

## 2 Methods

Our DFT calculations are performed primarily with the Vienna Ab Initio Simulation Package (VASP);^36−38^ we also use Quantum Espresso (QE).^39,40^ For VASP calculations, we use projector-augmented wave (PAW) potentials,^41^ treating 3d^2^ 4s^2^, 3d^10^ 4s^2^, and 2s^2^ 2p^3^ electrons explicitly for Ti, Zn, and N, respectively; for QE, we use optimized norm-conserving pseudopotentials (ONCVPSP v0.4) from PseudoDojo.^42^ We use the exchange–correlation functional of Perdew, Burke, and Ernzerhof (PBE)^43^ to compute total energies and Hellmann–Feynman forces to optimize the atomic structure. VASP is used for both the cation-ordered primitive cell and the cation-disordered supercells, and QE is used only for the cation-ordered unit cell. For the cation-ordered ZnTiN_2_ structure, both lattice constants and internal coordinates are relaxed. For cation-disordered supercells, we relax internal coordinates while keeping the lattice parameters fixed to those computed from the cation-ordered structure. A kinetic energy cutoff for the wave function of 600 eV is used for all VASP calculations, and 80 Ry (1088 eV) is used for all QE calculations. For these kinetic energy cutoffs, energy differences per atom are converged within 10^–5^ eV/atom in VASP and QE, and the fundamental band gap is converged within 1 meV for both VASP and QE. Total energies are converged to within 10^–7^ eV/atom, and all Hellmann–Feynman forces are below 0.01 eV/Å on each atom in our self-consistent VASP calculations. Total energies are converged to within 10^–5^ Ha, and all Hellmann–Feynman forces are below 10^–5^ Ha/Bohr on each atom in our self-consistent QE calculations. For our band gap and band structure calculations, we use the local density approximation (LDA), PBE, and four hybrid functionals, including the Heyd–Scuseria–Ernzerhof (HSE06) screened hybrid functional,^44,45^ PBE0,^46−48^ an optimally tuned global hybrid PBEα(0.147), and the Wannier optimally tuned screened range-separated hybrid (WOT-SRSH)^49^ functional. For the local density of states, the atomic orbital decomposition in the projected density of states utilizes the projector function provided by the PAW^41^ method in VASP.

As discussed below, we perform calculations for the cation-ordered crystal as well for one hundred disordered supercells. The cation-ordered wurtzite-derived structure has a *Pna*2_1_ space group and contains 4 formula units (f.u.), or 16 atoms, in the orthorhombic setting. Our cation-disordered 2 × 2 × 2 supercells (128 atoms) are based on the cation-ordered orthorhombic structure and include multiple Zn–Ti antisite defects. For DFT–PBE calculations, a Γ-centered 8 × 10 × 10 Monkhorst–Pack *k*-mesh is used for the 4 f.u. cation-ordered unit cell in VASP and QE, and a 4 × 4 × 4 *k*-mesh is used for all 32 f.u. cation-disordered supercells in VASP. For spontaneous polarization calculations using the modern theory of polarization,^50^ a 5 × 6 × 6 uniform Monkhorst–Pack *k*-mesh is used for the 4 f.u. cation-ordered interpolated structures between the ground state (*Pna*2_1_) and the hexagonal reference (*Pnma*) in VASP. For hybrid calculations, a 4 × 5 × 5 uniform Monkhorst–Pack *k*-mesh is used for the 4 f.u. unit cell in VASP and QE, and just the Γ point is used for most calculations of 32 f.u. supercells in VASP (a 2 × 2 × 2 or 4 × 4 × 4 *k*-mesh is used in hybrid calculations of 32 f.u. supercells for the density of states or time-dependent DFT in VASP). Total energy and band gap convergence with k-mesh density are shown in Supporting Information Tables S2–S4. In the calculation for formation energy, the configuration entropy is not included, and only the relative total energy is accounted for. Gaussian smearing is used in our Brillouin zone integrations, with a smearing parameter of 0.02 eV for structure relaxations and 0.05 eV in nonself-consistent calculations of the density of states. The ion-clamped dielectric tensor is obtained by calculating the self-consistent response of the system to finite electric fields with the HSE06 hybrid functional^44,45^ in VASP. The optical spectra are calculated using linear-response time-dependent DFT (LR-TDDFT) with VASP^51^ with PBEα(0.147) and a 4 × 5 × 5 *k*-mesh for the 4 f.u. cell (2 × 2 × 2 *k*-mesh for the 32 f.u. supercells). We provide the convergence of the optical spectra with the *k*-mesh for a select 32 f.u. supercell in Supporting Information Figure S13. For LR-TDDFT calculations with VASP and 32 f.u. supercells, 196 occupied bands below the VB edge and 150 unoccupied bands above the conduction band edge are included when solving the Casida equation.^52^ The complex shift used in the Kramers–Kronig transformation in these calculations is 0.10 eV.

## 3 Results and Discussion

### 3.1 Cation-Ordered ZnTiN_2_

We start with a discussion of a cation-ordered phase of ZnTiN_2_, the ground-state structure that has yet to be realized experimentally but provides useful context for what follows. Cation-ordered ZnTiN_2_ takes up a 16-atom (4 f.u.) wurtzite-derived orthorhombic primitive unit cell with space group *Pna*2_1_. Our calculated DFT–PBE lattice constants of the cation-ordered crystal are listed in Table 1. The ZnTiN_2_ structure can be viewed as a network of distorted nitrogen-centered corner-sharing tetrahedra with Zn and Ti at the corners. In the cation-ordered structure, the N–Zn_2_Ti_2_ (N22) tetrahedra can be viewed nominally as charge-neutral, obeying the octet rule:^26^ in an ionic limit, the average formal valence of the two Zn^2+^ cations and the two Ti^4+^ cations is 3+, precisely canceling the nominal charge of the N^3–^ anion. The tetrahedra are distorted, with larger N–Zn bonds of length 2.07 Å and smaller N–Ti bonds of length 1.94 Å. The bond lengths vary in supercells with cation disorder where antisite defects lead to charge-imbalanced tetrahedra (Supporting Information Figure S5). Compared to a competing cation-ordered structure with space group *Pmc*2_1_, which is composed of the same N22 motifs but where the Zn and Ti cations are arranged in a stripe pattern, the formation energy of the *Pna*2_1_ structure, in which the cations are more evenly distributed, is 16.4 meV/f.u. lower (Supporting Information Figure S1). Cation-ordered ZnTiN_2_ is polar and has a computed spontaneous polarization of 1.206 cm^-2^ along the crystallographic *c*-axis relative to a nonpolar hexagonal reference structure (Supporting Information Figure S2) where the cations and anions sit in the same plane, similar to values reported for cation-ordered ZnSnN_2_ (1.184 cm^-2^), ZnGeN_2_ (1.333 cm^-2^), and ZnSiN_2_ (1.433 cm^-2^).^27^ The large spontaneous polarization of ZnTiN_2_ suggests that polarization discontinuity may play a role near interfaces of a ZnTiN_2_ single crystal with other materials. The ion-clamped dielectric tensor of cation-ordered *Pna*2_1_ ZnTiN_2_ is obtained using DFT-HSE06 and a finite electric field approach,^53,54^ and our computed values of the diagonal components of ϵ_∞_^*xx*^, ϵ_*∞*_^*yy*^, and ϵ_*∞*_^*zz*^ are, respectively, 6.85, 6.65, and 6.92, the arithmetic mean of which is 6.81.

**Table 1:** Lattice Parameters of ZnTiN_2_ Calculated in VASP and of an Experimentally Synthesized Cation-Disordered ZnTiN_2_ Thin Film Measured by XRD^12^ and Calculated Dielectric Properites^a^

	lattice parameters (Å)			
lattice	DFT–PBE (ordered)	experiment (disordered)	dielectric tensor diagonal component	polarization (cm^-2^)
a	5.71	5.4	6.85	0
b	6.59	6.2	6.65	0
c	5.26	5.0	6.92	1.206

a Note: the experimental values are converted to an orthorhombic unit cell.

We compute the electronic structure of ZnTiN_2_ with DFT using different exchange–correlation functionals. Kohn–Sham DFT with semilocal functionals is known to underestimate the fundamental band gap,^55,56^ especially in semiconductors and insulators with d electrons.^57^ Semilocal functionals like PBE^43^ and LDA predict the fundamental band gap of cation-ordered ZnTiN_2_ to be 2.25 and 2.20 eV, respectively, values which are fortuitously close to the measured optical gap of the cation-disordered system, a result we can attribute to error cancellation. To obtain a more accurate prediction of the band gap, we use several hybrid functionals, including HSE06,^44,45^ PBE0,^46−48^ PBEα(0.147), and the recently developed WOT-SRSH functional.^49^ PBEα(0.147) is a global hybrid functional with the fraction of exact exchange set to 0.147, the inverse of the computed ZnTiN_2_ orientationally averaged high-frequency dielectric constant (6.81). WOT-SRSH is a range-separated hybrid functional with different fractions of exact exchange in the short and long ranges. The so-called range separation parameter, which dictates the length scale at which the Coulomb potential transitions from its short-range form to its long-range form, is tuned to satisfy an ionization potential (IP) ansatz;^58^ at long range, the fraction of exact exchange is set by the averaged high-frequency dielectric constant, here 6.81. WOT-SRSH has been shown to predict the fundamental band gap accurately for standard semiconductors,^49^ metal oxides,^59^ and halide perovskites,^60^ and it has been shown to be an optimal starting point for one-shot GW calculations.^61^ Using α = 0.25 (the fraction of exact exchange in short range) and α + β = 0.147 (the fraction of exact exchange in long range, determined by ), we determine that a range separation parameter of γ = 1.07 Bohr^–1^ satisfies the IP ansatz for cation-ordered ZnTiN_2_, following the procedure outlined in prior work^49^ (see Supporting Information for details).

All hybrid functionals considered predict a fundamental gap above 3.3 eV (Supporting Information Table S5), substantially larger than the PBE value and the optical gap reported experimentally. The predicted WOT-SRSH gap is 3.41 eV, which is only slightly larger than the HSE06 (3.37 eV) and PBEα(0.147) (3.31 eV) values. As mentioned, cation-ordered ZnTiN_2_ has not been synthesized in the experiments, and measurements have only been performed on the cation-disordered samples so far.^12,19^ However, our hybrid functional calculations suggest fundamental gap values of around 3.4 eV for the cation-ordered phase. A fundamental band gap of 3.4 eV for cation-ordered ZnTiN_2_ is 1.4 eV higher than the optically measured absorption onset of 2 eV. This large difference can potentially originate from multiple effects, including cation disorder, the presence of bound excitons, renormalization of the gap by phonons, and extrinsic defects. In what follows, we will focus on the role of cation disorder, which turns out to have a large effect, and, later, we will comment on excitons.

**Figure 1:**
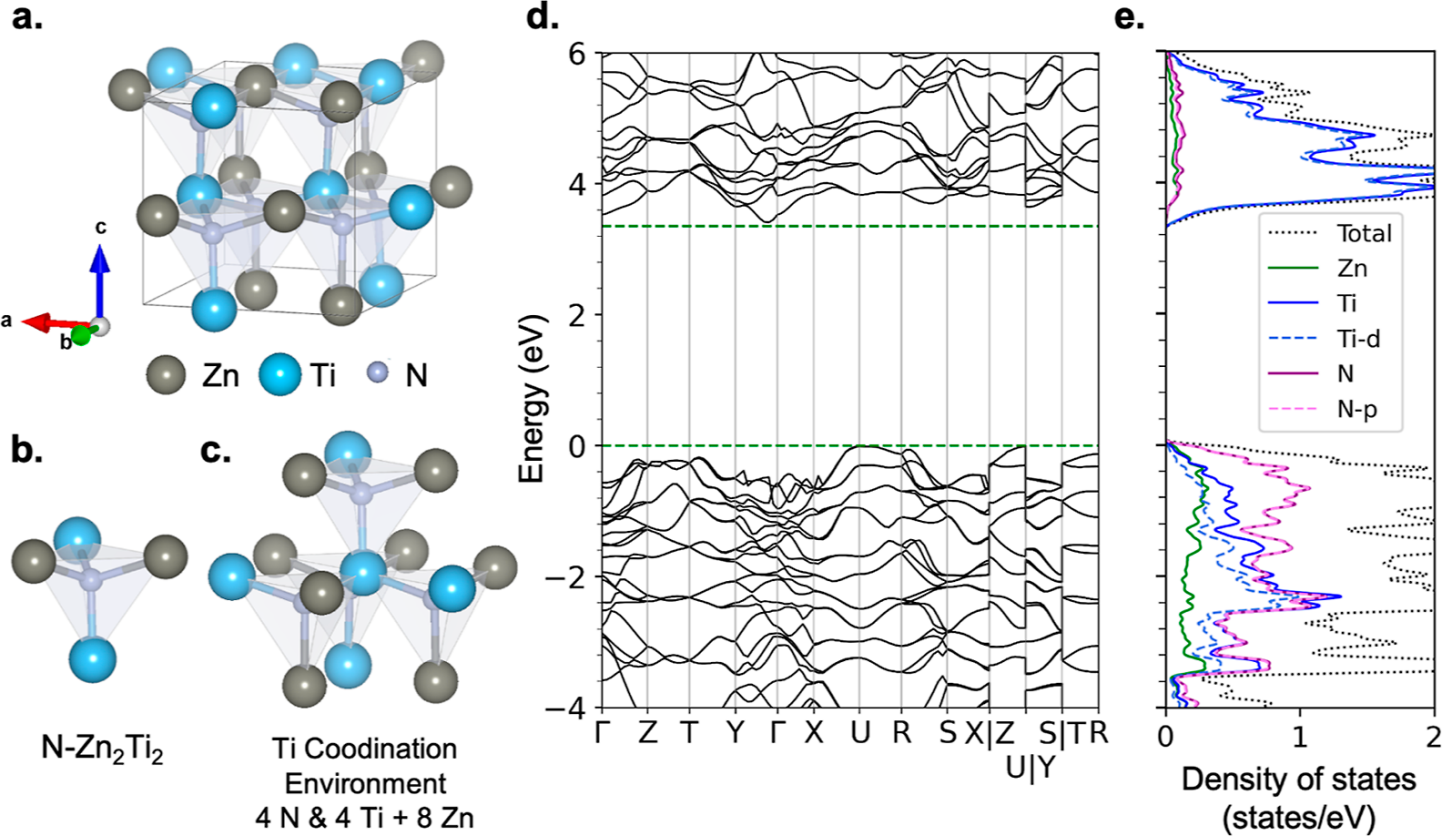
Atomic and electronic structure of cation-ordered *Pna*2_1_ ZnTiN_2_. (a) Unit cell atomic structure, with 16 atoms. (b) N-centered tetrahedral motif and local coordination of N by 2 Zn and 2 Ti atoms. (c) Local coordination environment of Ti, containing its nearest and next-nearest neighbors. (d) Electronic structure (DFT-HSE06), with energy relative to the valence band maximum (VBM). The indirect band gap is 3.35 eV. (e) Atom- and orbital-projected density of states. The color represents different species and orbitals. The purple dashed line and purple line almost overlap. (d) and (e) share the same *y*-axis.

Our computed DFT-HSE06 electronic structure of ZnTiN_2_ is shown in Figure 1d,e. The projected density of states of the cation-ordered phase shows N-p orbital-dominated valence band edges and Ti-d orbital-dominated conduction band edges, notably different from the more dispersive N-dominated conduction band edges of ZnGeN_2_^26,31^ and ZnSnN_2_.^21,26^

### 3.2 Cation-Disordered ZnTiN_2_

Despite the cation-ordered structure being energetically the most stable, cation disorder (via antisite defects) has been reported in the experimentally synthesized ZnTiN_2_ films,^12,19^ consistent with other wurtzite-derived zinc ternary nitrides.^4,29,34^ Cation disorder, with its higher configurational entropy, is hypothesized to occur because of relatively low formation energies of antisite defects^20−22^ and the limited kinetics during and after synthesis.^10,23^ As with other reported wurtzite-derived zinc ternary nitrides,^4,5^ the DFT band gap of the cation-ordered structure is overestimated compared to optical measurements, a difference that has been largely rationalized by cation disorder.^20,26,29,31,32^ Though the effects of cation disorder on the electronic structure for other zinc ternary nitrides have been noted before,^20,26,29−32,35^ the detailed manner in which cation disorder affects the band gap, and, in particular, the role of different possible local atomic structural motifs, is not well understood. While Si and Ge would assume 4+ stable nominal oxidation states, Sn is a heavier element with two stable nominal oxidation states (2+ and 4+). Ti features both d and s valence electrons and a 4+ oxidation state, leading to d orbital-dominated conduction bands. With increasing degrees of cation disorder, ZnSnN_2_ shows a gradual band gap reduction of the order of 1 eV with moderate band edge localization, and band gap closure has been reported in randomly distributed cation-disordered supercells in a previous theoretical investigation.^32^ For ZnGeN_2_, calculations predict that a high density of cation disorder leads not only to a large band gap reduction and metallic behavior (which is not observed in the experiment) but also to significant localization at the band edges.^31^ Since ZnTiN_2_ has conduction band edges with a Ti d character, cation disorder is likely to affect the band gap and near-edge band structure differently relative to ZnSnN_2_ and ZnGeN_2_.

Prior studies of cation disorder in ternary nitrides have been predominantly computational since it is challenging to measure and manipulate the degree of disorder in experiments.^10,24^ In DFT-based computational studies, one constructs a supercell and introduces disorder by creating antisite defects through random or targeted cation swaps. In generating supercells, cation disorder has been accounted for in three main distinct ways in prior work: via the special quasirandom structure (SQS) method,^18^ using Monte Carlo (based on first-principles energetics) to probe possible configurations at various temperatures,^30−32,35^ and introducing a fraction of low cation disorder via deliberate antisite defect placement.^20,26^ SQSs^62^ mimic a nonperiodic disordered system in a relatively small supercell to save computational cost. However, previous use of SQSs led to the erroneous prediction of metallicity in ZnSnN_2_^32^ and a significantly underestimated band gap of ZnGeN_2_^20^ as heterovalent ternary nitride systems with different cation sizes do not distribute randomly.^32^ Previous studies using Monte Carlo simulations^30−32,35^ reported changes in the degree of cation disorder with increasing temperature and provided equilibrium configurations for different temperatures. In such simulations, an accurate configuration space sampling requires constructing a suitable model Hamiltonian, which is dependent on the material. For example, ZnGeN_2_ cannot be well described by a local motif-based model Hamiltonian, unlike ZnSnN_2_; thus, a more complex cluster-expanded Hamiltonian for ZnGeN_2_ was required.^30^ By applying cation swaps to create different possible cation-disordered supercells, previous first-principle calculations^20,26^ demonstrated that cation-disordered structures containing only octet-rule-conserving motifs have energies and electronic structures similar to those of the cation-ordered structure, emphasizing the effect of octet rule-violating motifs. However, only a few specific supercells with a small number of octet-rule-violating motifs were considered in prior first-principles studies, leading to an incomplete understanding of band gap reduction in such systems. In what follows, we construct multiple supercells, including the five possible N-centered tetrahedra (and multiple arrangements of them), with low-density, high-density, and random cation disorder, to understand the consequences of cation disorder in ZnTiN_2_. Further, we demonstrate quantitatively how the band gap is reduced in cation-disordered supercells and provide an intuitive mechanism which is supported by our calculations and can be extended to other zinc ternary nitrides.

#### 3.2.1 Cation-Disordered ZnTiN_2_ Supercells

We construct 100 supercells, each with 32 f.u. (128 atoms) having different degrees of cation disorder. The cation disorder is introduced by interchanging several Zn and Ti cations to create antisite defects. We limit the antisite defects, or swaps between Zn and Ti cations, to neighbors only. Swapping neighboring Zn and Ti atoms can introduce four possible octet-rule-violating N-centered tetrahedral motifs, and we denote these different tetrahedra as N–Zn_4_Ti_0_ (N40), N–Zn_3_Ti_1_ (N31), N–Zn_1_Ti_3_ (N13), and N–Zn_0_Ti_4_ (N04). We will use the number of octet-rule-violating motifs in supercells with a total of 64 N-centered tetrahedra to quantify the density of cation disorder. Compared with N13 and N31, N40 and N04 violate local charge neutrality to a greater degree, suggesting a higher energy cost; as will be discussed below, this is indeed the case, especially for N40. The supercells considered in our work contain mostly N22, N13, and N31 motifs and relatively fewer N40 and N04 motifs (if the 64 cation sites are perfectly randomly occupied or alloyed by 50% Zn and 50% Ti, the concentrations for N40, N31, N22, N13, and N04 would be, respectively, , , , , and .).

Of our 100 supercells, 18 supercells have what we define as low cation disorder, featuring between 9%  and 14%  octet-rule-violating motifs; 80 are high cation-disordered supercells with 48%  to 57%  octet-rule-violating motifs; one supercell has cations that are randomly occupied by Zn or Ti with the Zn:Ti = 1:1 stoichiometry fixed (called Supercell *R*); and one supercell introduces additional antisite defects to Supercell *R* to eliminate N40 and increase N22 motifs (called Supercell R*).

The 18 low cation-disordered supercells feature different low-density configurations of octet-rule-violating motifs. For example, all four possible configurations with 9% octet-rule-violating motifs are included here; analyzing these supercells allows us to examine changes in the electronic structure due to different atomic arrangements at a fixed cation disorder density. In the low-cation disorder regime, octet-rule-violating motifs are correlated since they are related by one or two swaps, whereas in supercells with high cation disorder, such correlations are absent. To generate supercells with high cation disorder, we randomly add 1 or 2 different antisite defects to a supercell with 50% N22 and generate the rest of the 79 supercells. With the 128-atom supercell size, 1 or 2 swaps can influence 9.4–18.8% motifs, enough to create meaningful changes in the motif arrangement. We focus on supercells with 48–57% octet-rule-violating motifs because the cation disorder density remains similar after an additional swap in this range (in contrast, the degree of cation disorder generally increases with just a single additional antisite defect in the low-cation disorder range). Supercell *R* models ZnTiN_2_ in the solid solution limit, providing a useful reference. Starting from Supercell *R*, Supercell R* removes energetically unfavorable N40 tetrahedra while retaining a high degree of cation disorder. A comparison of results for Supercell *R* and R* therefore specifically highlights the effects of N40 motifs on the high-cation disorder limit. Our approach to constructing cation-disordered supercells and further details of our calculations are in the Supporting Information.

Supercells with an intermediate amount of cation disorder, 14–48% octet-rule-violating motifs, are not considered here. Their formation energies and electronic structures could be extrapolated from the general linear trend in energetics discussed in Section 3.2.2 and the band gap reduction mechanism described in Section 3.3. By examining how the short-range charge imbalance affects the energetics, electronic structure, and optical properties in our supercells, we can infer likely local atomic configurations and the corresponding properties of the experimental systems.

All constructed 128-atom (64 N motifs) supercells are internally relaxed at fixed lattice constants using DFT–PBE. The choice of fixed lattice parameters is discussed in the Supporting Information, where the relaxation of lattice parameters in instances is found to change the lattice constants and fundamental band gap by a negligible amount.

#### 3.2.2 Cation-Disordered Energetics

**Figure 2:**
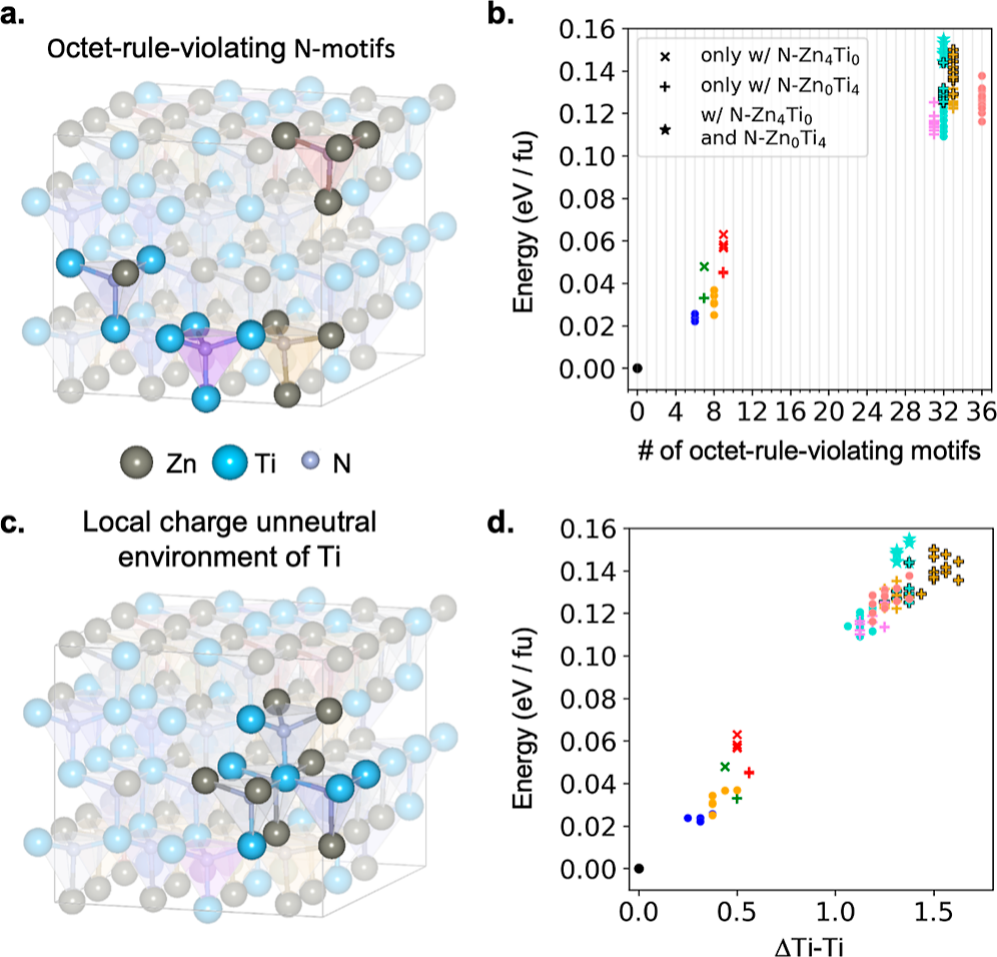
(a) 4 types of octet-rule-violating motifs in a cation-disordered supercell. (b) Relative energy versus the number of octet-rule-violating motifs. (c) Local charge-unneutral Ti environment in a cation-disordered supercell. (d) Relative energy versus ΔTi–Ti, the spatially averaged deviation of the number of Ti–Ti neighboring pairs in the Ti local environment in a supercell. × indicates the presence of N–Zn_4_Ti_0_; + indicates the presence of N–Zn_0_Ti_4_; ★ indicates the presence of both N–Zn_4_Ti_0_ and N–Zn_0_Ti_4_. Black outlines indicate that relatively more N–Zn_0_Ti_4_ motifs existed in the supercell. The same symbol (both color and shape) represents the fact that each kind of motif in these supercells has the same density. The structure details of supercells are mentioned in Table 2 and the Supporting Information.

In Table 2 and Figure 2, we summarize our calculations of the total energies of all supercells considered relative to cation-ordered ZnTiN_2_, with their different degrees of cation disorder. All total energies are obtained from DFT–PBE calculations. Motif densities of low-cation-disordered Supercells 1–12 and high-cation-disordered Supercells *R* and R* are listed in Table 2. Among Supercells 1–4 (all of which contain  N22 motifs), Supercell 3 has the lowest energy and Supercell 4 has the largest but by only 3.5 meV per f.u. These four supercells feature the lowest cation disorder density among our 100 128-atom supercells. In these supercells, the octet-rule-violating motifs are proximal to each other, and the arrangements of motifs are similar, resulting in small energy differences.

**Table 2:** Energy Relative to the Cation-Ordered Limit and Band Gap of Selected Low and Randomly Occupied Cation-Disordered ZnTiN_2_ Supercells^a^

no.	N40	N31	N22	N13	N04	ΔTi–Ti	energy/f.u. (meV)	*E*_*g*_^PBE^ (eV)	VBM shift (eV)	CBM shift (eV)	*E*_*g*_^PBEα(0.147)^ (eV)
1	0	3	58	3	0	0.3125	23.7	1.98	0.14	0.13	3.10
2	0	3	58	3	0	0.25	23.8	1.98	0.13	0.13	3.10
3	0	3	58	3	0	0.3125	22.2	1.94	0.18	0.13	3.10
4	0	3	58	3	0	0.375	25.7	1.81	0.17	0.26	2.91
5	1	2	57	4	0	0.4375	48.0	1.43	0.70	0.11	2.53
6	0	4	57	2	1	0.5	33.0	2.02	0.12	0.11	3.20
7	0	4	56	4	0	0.4375	36.7	1.71	0.23	0.31	2.81
8	0	4	56	4	0	0.375	34.2	1.79	0.23	0.23	2.89
9	0	4	56	4	0	0.375	31.0	2.05	0.09	0.11	3.18
10	0	4	56	4	0	0.5	36.9	1.91	0.21	0.13	3.02
11	0	4	56	4	0	0.375	30.5	2.08	0.14	0.03	3.21
12	0	4	56	4	0	0.375	25.0	2.12	0.08	0.05	3.35
*R*	4	17	21	19	3	2.1875					0.67
R*	0	25	19	15	5	1.9375	215.4	1.13	0.57	0.55	2.12

a VBM is the abbreviation for valence band minimum, and CBM is the abbreviation for conduction band maximum. ΔTi–Ti, defined in eq 1, is a descriptor for the short-range charge imbalance of the cation environment.

Comparatively, Supercells 5 and 6 (with  N22 motifs) have a larger formation energy per octet-rule-violating motif, especially Supercell 5. The relative energies of Supercells 5 and 6 are 48 and 33 meV per f.u., respectively, compared to cation-ordered structures, the first of which has a larger energy than Supercells 7–12 ( N22 motifs), which contain more octet-rule-violating motifs. N40 evidently has a large formation energy, followed by that of N04. Supercells 13–18 ( N22 motifs), which contain N40 or N04 motifs, confirm this finding (Supporting Information Table S6). Therefore, N04 and N40 have asymmetric energy penalties, which originates from different Zn and Ti cation chemistry and is consistent with the predicted thermochemical stability of the Zn–Ti–N system,^12^ where Zn_3_N_2_ has a higher formation energy relative to TiN.

Compared to Supercells 1–4, the energies of Supercells 7–12 increase by an average of 8.5 meV per f.u. due to the additional N13 and N31 motifs. Because the distribution of octet-rule-violating motifs is different in each supercell, the formation energy modestly varies. The atomic arrangements within these supercells clearly suggest that the size of a 128-atom supercell is not large enough to view octet-rule-violating motifs in the dilute limit (Supporting Information Figures S10 and S11). The energetics include the interactions among octet-rule-violating motifs and their images under periodic boundary conditions. Supercells 7 and 8 have N13 (or N31) motifs clustering together, and the high formation energies of these supercells reflect the relatively high short-range charge imbalance. In Supercell 12, every N13 (or N31) motif has a N31 (or N13) neighbor. With the neutralizing effect of adjacent N13 and N31 motifs, the system exhibits a lower degree of short-range charge imbalance and a lower formation energy.

Figure 2 summarizes the relative formation energies of both low- and high-density cation-disordered supercells, compared with a cation-ordered crystal. In Figure 2, data points in the same color indicate that the supercell has the same total number of charge-imbalanced octet-rule-violating motifs and data points in the same shape and color indicate the same numbers of each type of octet-rule-violating motif (N31, N13, etc.). Although supercells with the same numbers of total octet-rule-violating motifs have different energies, originating with the variety of motifs and their arrangements, the general trend is that more octet-rule-violating motifs lead to a higher average formation energy per f.u. The standard deviation of relative energy difference in supercells with the same number of each type of octet-rule-violating motif is less than 6.2 meV per f.u., indicating that the numbers of each type of octet-rule-violating motif can be used to roughly estimate the formation energy of a cation-disordered structure. The energy per octet-rule-violating motif is similar among non-N04- or -N40-containing supercells, as shown in Supporting Information Figure S6. With more N04 motifs, the average formation energy generally increases, as shown in the comparison between the + marker with (more N04) and without a black edge. With N40 motifs, the average formation energy is significantly higher than that of other supercells containing the same number of total octet-rule-violating motifs. However, we cannot exclude these motifs from being present in the experimentally synthesized systems due to their high formation energies since cation disorder is likely stabilized by limited atomic kinetics during synthesis. Nevertheless, the understanding of the formation energy trend with cation disorder is still important for the future engineering of cation disorder.

The energetic cost of the random structure (Supercell *R*) is not included in this analysis since PBE predicts that the system is metallic (although a band gap exists for this supercell using our chosen hybrid functionals). Supercell R* is 215.4 meV per f.u. higher than the cation-ordered cell, a large relative formation energy resulting from its many local charge-imbalanced motifs.

In summary, the energetics of cation-disordered supercells can be understood in terms of short-range charge imbalance, namely, the local environment of anions and cations. The local environment of the N anion is quantified by its nearest neighbors, which are the corner atoms of the N motif (Figure 1b). As shown in Figure 2, when the local environment of a large proportion of N atoms is charge-imbalanced, that is, when the number of octet-rule-violating motifs increases, the energetic cost per f.u. rises.

We define the spatially averaged deviation of the number of Ti–Ti neighboring pairs in the Ti local environment relative to a cation-ordered cell, ΔTi–Ti, as a descriptor for the short-range charge imbalance of the cation environment. For cation-ordered ZnTiN_2_, the second-nearest neighbors of each Ti consist of 8 Zn and 4 Ti atoms (Figure 1c); thus, the number of Ti–Ti neighbors is 4. Figure 2c shows one Ti’s neighboring atoms up to the second-nearest neighbor in vivid colors in a cation-disordered supercell, where the number of Ti–Ti pairs is 5. We define
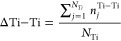
1, where *n*_j_^Ti-Ti^ is the number of Ti-Ti pairs in *j* th Ti local environment and *N*_Ti_ is the number of Ti atoms in the supercell. ΔTi–Ti is correlated with the number of octet-rule-violating motifs: a greater number of octet-rule-violating motifs generally increases ΔTi–Ti. The presence of N04 and N40 motifs also increases ΔTi–Ti. ΔTi–Ti provides a measure of the overall short-range charge imbalance of the cation environment beyond the nearest neighbors. Of course, ΔTi–Ti does not capture the spatial distribution of the same cation species. In addition, ΔTi–Ti, being an average value, does not reveal the detailed nature of the local cation clustering in the supercell. However, as shown in Figure 2d, the average relative energy in cation-disordered supercells is strongly correlated with ΔTi–Ti.

### 3.3 Electronic Structure and Band Gap of ZnTiN_2_

Our band structure and density of states for cation-ordered ZnTiN_2_, computed with DFT, appears in Figure 1. The cation-ordered system exhibits a fundamental band gap of 3.4 eV with DFT-WOT-SRSH. The valence band edge has a predominantly N 2p character, and the conduction band edge is principally of Ti 3d character. As discussed above and elsewhere,^20,26,29,31,32^ cation disorder reduces the band gap. As was first postulated in our prior work,^12^ the charge-imbalanced environment leads to local changes in the electrostatic potential that result in a relative shift of state density at the valence and conduction band edges, ultimately narrowing the gap. In what follows, we elucidate this mechanism for band gap reduction in detail and, in particular, illustrate how the gap reduction depends on the number and distribution of antisite defects.

**Figure 3:**
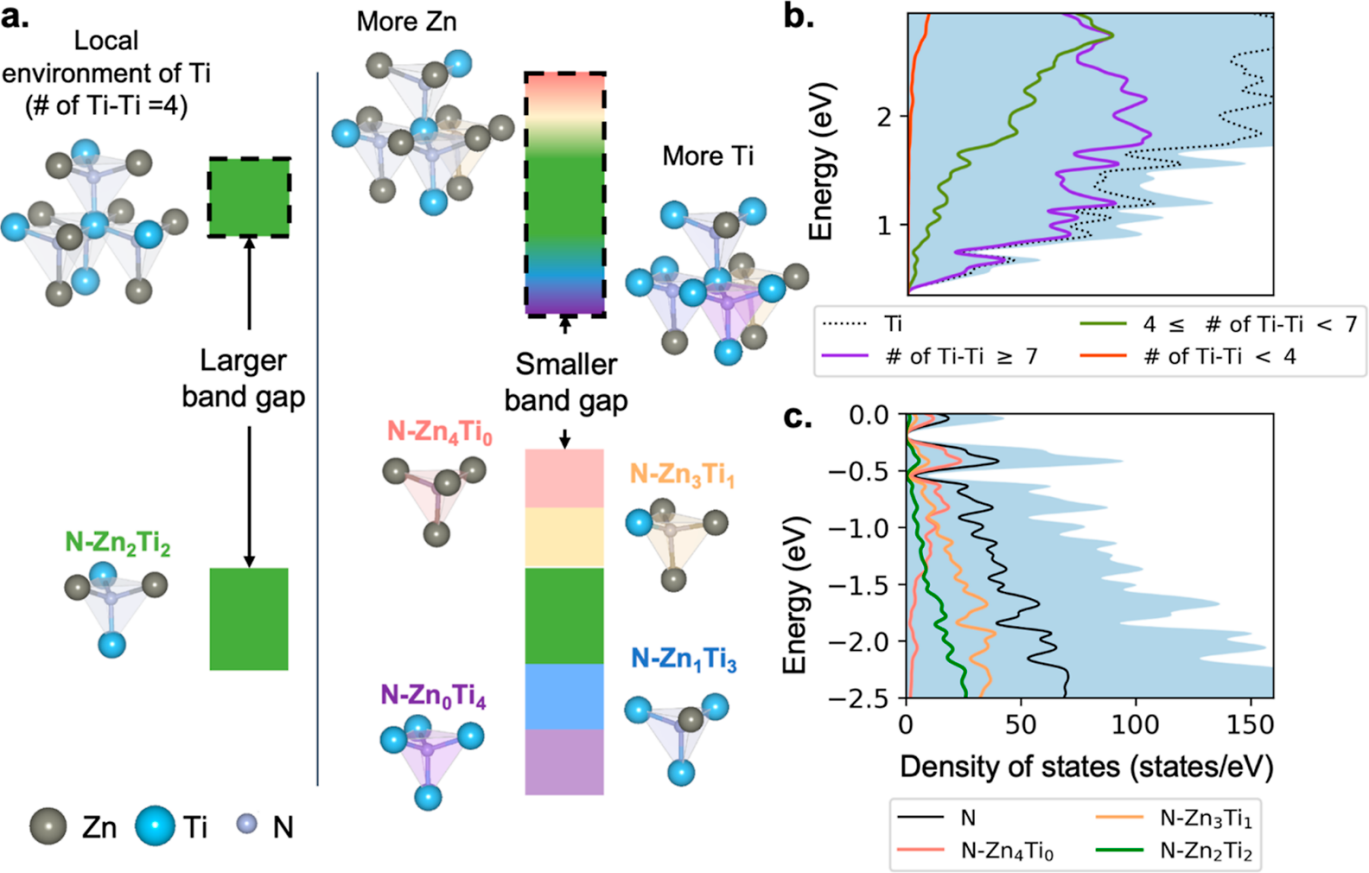
(a) Scheme of band gap reduction by valence and conduction band broadening induced by cation disorder. The colored rectangle indicates bands; those rectangles with a dashed black edge indicate conduction bands. The color of the bands suggests the positive (blue–purple) or negative (pink–orange) local environment. The left panel is the cation-ordered case; the right panel is cation-disordered. (b) Ti projected density of states of conduction bands in Supercell *R*. Different colors correspond to different numbers of Ti–Ti neighboring pairs (# of Ti–Ti) in such a Ti local environment. (c) N projected density of states in Supercell *R*. Different colors correspond to N atoms in different motifs.

Since the ZnTiN_2_ valence band edges are dominated by the N-p orbital character, we consider how the local environment of different N motifs influences the valence band edge energies. For the N40 and N31 motifs, the corner atoms of the N motifs are less positively charged compared with those of N22 (since there are more Zn^2+^ and less Ti^4+^ cations), suggesting a smaller electron binding energy locally that would shift N p-dominated bands upward in energy. Accordingly, the upward shift in the valence band edge observed for cation-disordered supercells is associated with N40 and N31 motifs (as shown via the partial density of states of those motifs in Figure 3c). In particular, the N40 motif, which would have the smallest electron binding energy, dominates the top of the valence band. Conversely, contributions to the valence band from N13 and N04 motifs shift to lower energies. In this way, we can see those octet-rule-violating N-center motifs—which come in pairs that are net negative and positive—act to broaden the valence band, moving the valence band maximum (VBM) to a higher energy relative to the ordered structure.

Similarly, an analysis of the local environment of Ti can explain the effects of cation disorder on the conduction band, which is predominantly of a Ti 3d character. The nearest neighbors of Ti are all N anions, so we use the ΔTi–Ti descriptor to describe its local chemical environment. Conduction bands with a character associated with Ti cations having more Ti second-nearest neighbors are shifted to lower energies since the electrons experience a more attractive electrostatic potential near these Ti cations. As with N and the valence bands, the local charge imbalance around Ti cations broadens the conduction bands, resulting in shifts of some of the conduction bands to lower energies and some to higher energies.

In summary, cation disorder creates short-range charge imbalance that broadens valence band and conduction band width, resulting in a smaller band gap (shown in Figure 3a). More short-range charge imbalance has a higher associated energetic cost but causes a larger band gap reduction. The DOS (DFT-HSE06) of Supercell *R*, the randomly occupied cation-disordered supercell, is shown in Figure 3b,c, with conduction band and valence band edges projected on the different Ti and N local environments, respectively. N40 motifs dominate the valence band maxima, and the N31 motifs dominate the valence bands near the maxima. Ti cations with more Ti neighbors play an important role in decreasing the conduction band edges. We note that this physical picture conforms to the standard intuition that the isolated substitutional Ti defect, Ti_Zn_, is a donor and the substitutional Zn defect, Zn_Ti_, is an acceptor.

DFT–PBE is used to calculate the electronic structure of cation-disordered supercells for computational efficiency. The use of PBE incorrectly enhances the interaction between d orbitals of Zn and Ti and p orbitals of N, and, overall, it is expected to underestimate the fundamental gap. Therefore, the calculated band gap should be viewed as qualitative and not quantitative. For comparison, we perform calculations using the tuned global hybrid functional PBEα(0.147) in all considered supercells. The obtained average difference between the PBEα(0.147) and PBE band gaps is 1.07 eV (standard deviation of 0.05 eV), close to the difference in the cation-ordered cell. The band gap difference between PBE and PBEα(0.147) suggests that nonlocal Fock exchange does not change the nature of the band-shifting mechanism and our focus on the relative PBE band edge shifts is adequate.

**Figure 4:**
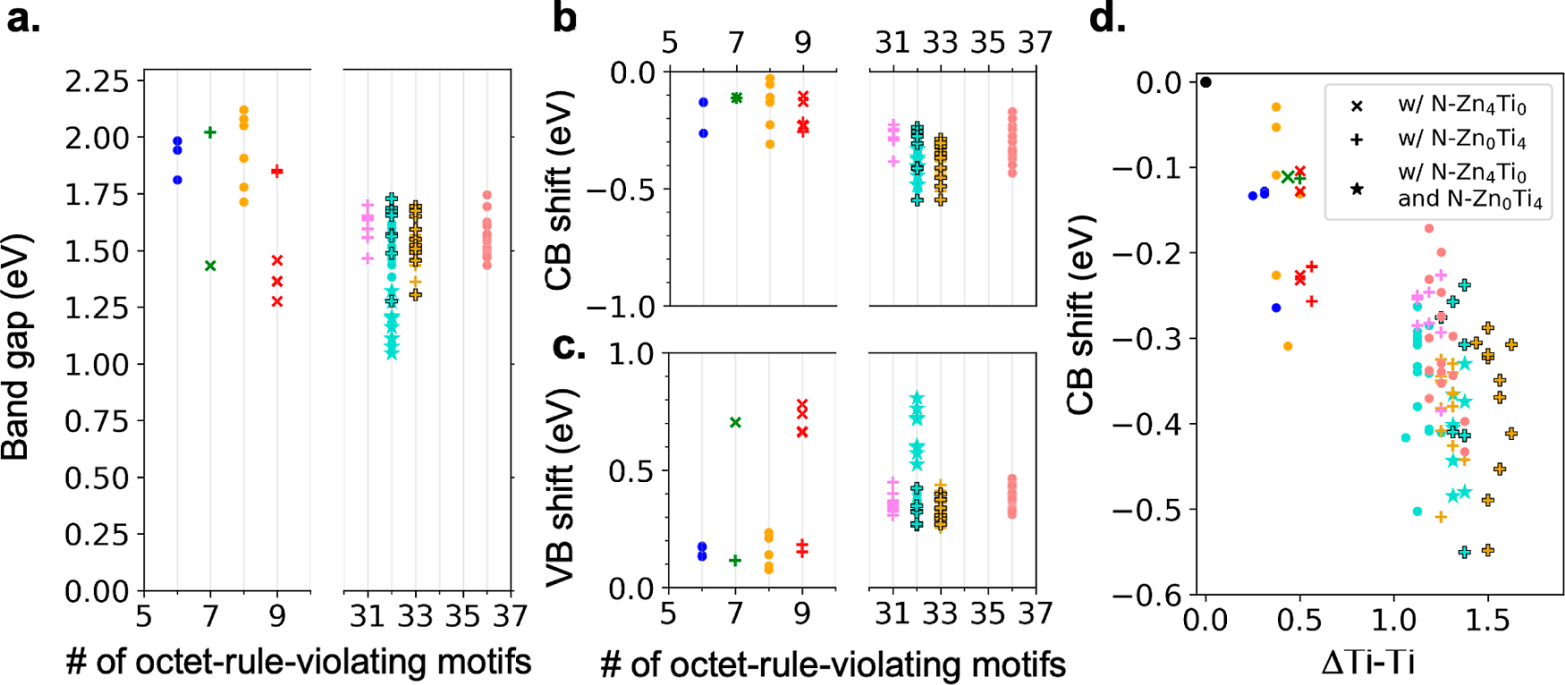
Band gap change and band edge shift in cation-disordered supercells. The color representation is the same as in Figure 2. (a) DFT–PBE band gap change versus the number of octet-rule-violating motifs. (b) conduction band (CB) and (c) valence band (VB) edge position shift, relative to the cation-ordered cell. (d) CB edge position shift versus ΔTi–Ti (defined in eq 1).

Our calculated band gap and band edge energies of all supercells are summarized in Figure 4 and Table 2. Both low- and high-cation-disordered supercells have band gaps in the range of 1.05–2.05 eV (DFT–PBE). The band edge energies in Figure 4 are reported with respect to the cation-ordered crystal. The band gaps vary in supercells with the same number of each type of octet-rule-violating motifs (indicated by the same color and symbol shape) because of different antisite defect arrangements. Supercells with non-N40 and -N04 motifs show a general trend of a decreasing band gap with an increasing number of antisite defects, but this trend in band gap reduction seems to be saturated in the high cation disorder density.

In low-cation-disordered supercells, octet-rule-violating motifs are introduced by one or two cation swaps, and their spatial proximity and common origins closely couple them. For supercells with a low density of cation disorder, the band edge states are largely localized on charge-imbalanced motifs, which indicates that the arrangement of imbalanced motifs can cause moderate changes in localized band edge energies. For example, the band gaps of Supercells 7–12 differ within 0.5 eV due to their distinctive defect arrangements. Supercells with energetically more costly N40 motifs exhibit a band gap reduction of almost 1 eV because the valence band maxima are dominated by N-orbital-derived sites associated with these extremely charge-imbalanced motifs. Our low-cation-disordered supercells with N04 motifs do not have particularly small gaps because the most positive local charge imbalance near Ti cations is alike compared with other low-cation-disordered supercells without N04 motifs, which is inferred from the unchanged value of the maximum number of Ti–Ti neighbors.

Our highly cation-disordered supercells generally have lower band gaps than those of low-cation-disordered supercells without N40 motifs. From Figure 4b,c, for those without N40 motifs, the distribution of valence band edge shifts is smaller than that of conduction band edges in these cases; supercells with N40 motifs exhibit larger valence band edge shifts. Supercells with N04 motifs are more likely to have larger conduction band edge shifts due to enhanced short-range charge imbalance near Ti atoms. Figure 4d shows that the conduction band edge downshift generally increases as the ΔTi–Ti bond becomes larger. The varieties of conduction band edges at the same ΔTi–Ti suggest that ΔTi–Ti cannot fully capture the extreme short-range charge-imbalanced Ti environment, as stated earlier.

As shown in Figure 4a, the band gap reduction is not necessarily monotonic with the number of octet-rule-violating motifs. The motifs resulting in an extreme short-range charge imbalance largely determine the band edge shifts. In those high-cation-disordered supercells without N40 motifs, the average computed valence band edge shifts are around 0.36 eV (standard deviation 0.05 eV) and conduction band edge shifts are around 0.34 eV (standard deviation 0.08 eV), as determined with DFT–PBE. We note that the experimental ZnTiN_2_ films may have a higher or lower degree of cation disorder than the above-mentioned high cation disorder range or contain more energetically unfavorable motifs due to limited kinetics during synthesis, which can lead to different valence and conduction band edge shifts. If experimental systems contain a higher degree of disorder, then whether the conduction band edge continuously downshifts depends on the existence of a larger short-range charge imbalance of the Ti environment than those in our high-cation-disordered supercells. If there are no N40 motifs in these supercells, the small change in valence band edge energy depends on the interactions between N31. Supercell R*, which has more N31 motifs than N22, is an example, where the valence and conduction band edge shift are 0.57 and 0.55 eV, respectively. The DFT–PBEα(0.147) band gap of Supercell R* is 2.1 eV, close to the optical onset. If the experimental samples contain more N40 motifs, their valence band edge states can be higher in energy, at the expense of being localized, and potentially act as midgap states that can trap carriers, as discussed in Section 3.4.

### 3.4 Density of States and Carrier Localization

We examine the localization of states at the band edge and the density of states of our supercells. To examine the relative spatial localization of states, we use the inverse participation ratio (IPR),^32^ defined as
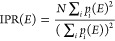
2where *E* is the energy, *p*_*i*_ is the local density of states projected on atom *i* in the supercell, and *N* is the total number of atoms in the supercell. This ratio quantifies the localization degree of states at a given energy *E*: for a perfectly delocalized state at a given energy *E*, IPR(*E*) = 1; for a state localized on only one atom, IPR(*E*) = *N*.

**Figure 5:**
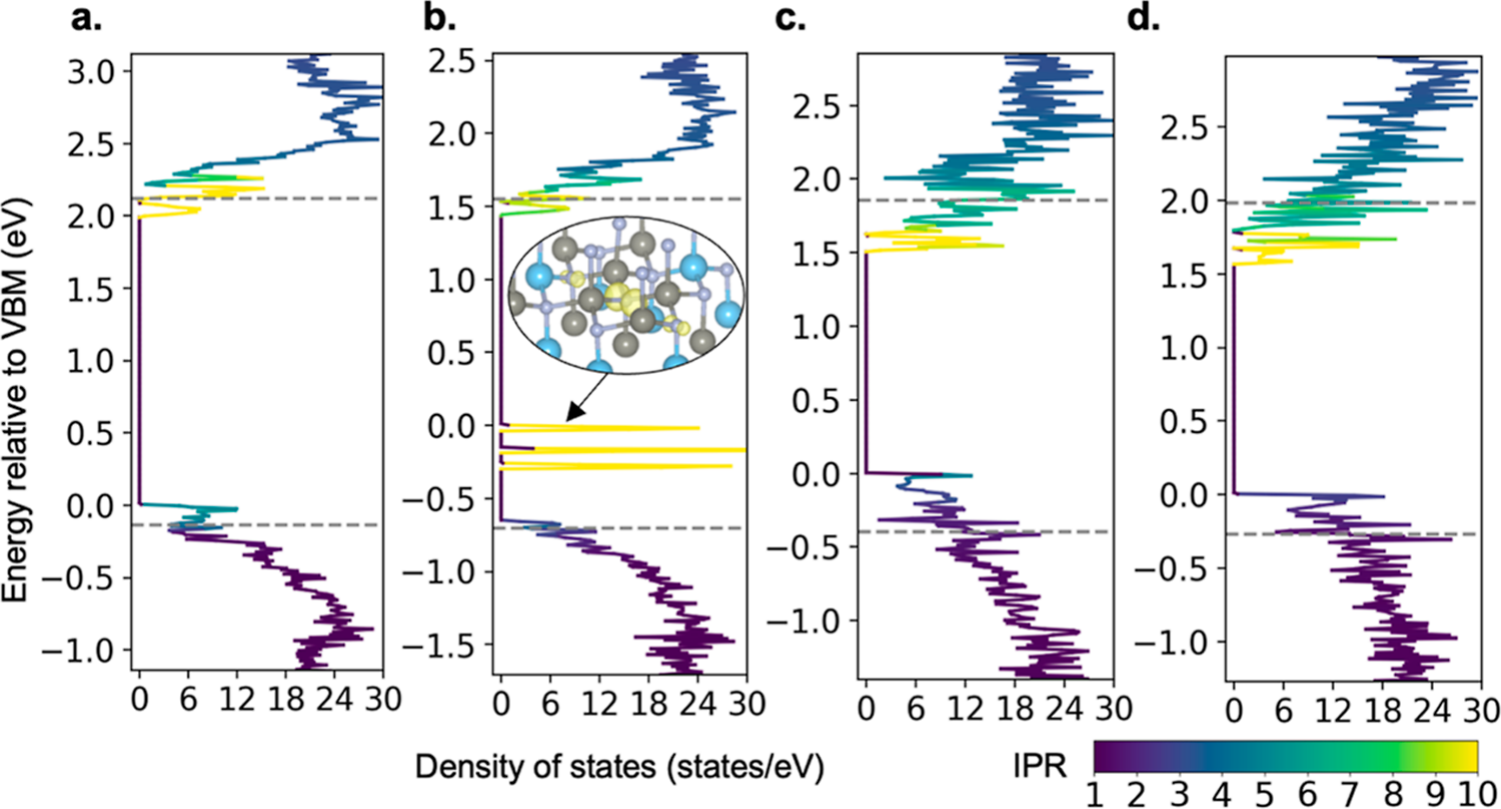
Density of states with the IPR value colored in cation-disordered supercells. (a) Supercell 1 (0 N40,  N31,  N22,  N13, and 0 N04) (b) Supercell 5 ( N40,  N31,  N22,  N13, and 0 N04), (c) a high-cation-disordered supercell with 0 N40,  N31,  N22,  N13, and 0 N04, and (d) a high-cation-disordered supercell with 0 N40,  N31,  N22,  N13, and  N04. The charge density isosurface (10% of maximum value) of the highest occupied valence band is shown in (b), which is centered on the only existing N40 in the supercell, and many atoms are not shown in the structure for simplicity. The number of each type of octet-rule-violating motif in the supercell is listed in the text. The gray dashed lines indicate the valence and conduction band edges in cation-ordered ZnTiN_2_. The *y*-axis is the energy relative to the VBM in each supercell.

The densities of states of two low- and two high-cation-disordered supercells with associated IPRs are shown in Figure 5. The conduction band edges of ZnTiN_2_ have higher IPR values than those of valence bands due to the large contribution of Ti-d orbitals to conduction band edges (the IPR for cation-ordered ZnTiN_2_ is in Supporting Information Figure S4 for comparison). The dashed lines in Figure 5 show the estimated relative band edge energy of cation-ordered ZnTiN_2_ to visualize the band edge shifts due to cation disorder. The IPR values of the bands beyond the cation-ordered band edge level are similar among the different supercells. The high IPR values for states at the band edge are closely related to the short-range charge-imbalanced environment caused by cation disorder. The IPR at conduction band edges is consistently high among these four supercells since the conduction band edges are dominated by a few Ti atoms with the most positive local environment.

In low-cation-disordered supercells (Figure 5a,b), there are a limited amount of negatively charge-imbalanced N40 and N31 motifs. These motifs lead to high IPR values of valence band edges. Figure 5b shows sharp peaks at valence band edges, which are dominated by the N in the center of the one N40 motif. Similarly, those few Ti atoms with short-range charge imbalance are responsible for the character of the states at the conduction band edges, leading to localized conduction band edges. Since N40 motifs create localized states that can be viewed as deep in-gap states, reducing N40 motifs would be critical to enhancing carrier transport in semiconducting ZnTiN_2_.

However, in high-cation-disordered supercells (Figure 5c,d), charge-imbalanced N motifs dominate, and the density of near-valence band-edge states increases. The interaction between negatively charged octet-rule-violating N motifs increases, so the near-valence band-edge states are more delocalized (shown in Supporting Information Figure S8). Therefore, the IPR values near the valence band maxima in Figure 5c are lower than those in Figure 5a,b. The correlation between N31 motifs indicates that the hopping of hole carriers is not strongly hindered as long as there are relatively few or no N40 motifs. On the other hand, Ti-d orbitals are more localized compared with N-p orbitals. Despite the increased number of Ti cations experiencing a charge-imbalanced environment that causes the increased density of near-conduction band-edge states, the conduction band edges are contributed by the Ti atoms with the most positive charge-imbalanced environments, which are still more localized than the valence bands. The high IPR values of states near conduction band edges associated with a Ti character suggest relatively low electron carrier mobility in ZnTiN_2_, unlike ZnSnN_2_,^32^ which may be partly responsible for the low electron mobility of prior ZnTiN_2_ samples, along with oxygen impurities.^12^

### 3.5 Cation Disorder-Induced Band Gap Reduction in Other Ternary Nitrides

Our understanding of the atomic-scale origin of band gap reduction can be extended to other similar zinc ternary nitrides such as ZnGeN_2_ and ZnSnN_2_. The N p orbital-dominated valence bands are broadened via the same explanation as that in Section 3.3. Previous theoretical studies^20,31,32^ have found the dominance of states with characters derived from N–Zn_4_Ge_0_ and N–Zn_3_Ge_1_ (or N–Zn_4_Sn_0_ and N–Zn_3_Sn_1_) motifs near valence band edges in ZnGeN_2_ (or ZnSnN_2_). Both ZnGeN_2_ and ZnSnN_2_ have conduction band minima with a N s-orbital character.^22,27^ Cation disorder-induced local charge imbalances can cause electrostatic potential differences for N s states, as well, thus broadening the bandwidth of the N s orbital band. Despite the increased bandwidth, the delocalized N s states do not result in a significant increase in the IPR near the conduction band minimum,^32^ unlike the previously observed IPR enhancement near valence band edges, which are dominated by more localized N 2p orbitals. Prior work^31^ on ZnGeN_2_ has reported Zn-contributing high IPRs for states separated from conduction bands in some high-cation-disordered supercells, which seems to be in disagreement with this mechanism. A prior work used a single-shot hybrid functional approach with DFT + *U* wave functions fixed to obtain band structures at the Γ point since its large 1024-atom supercell size prevents the use of self-consistent hybrid calculations or more advanced many-body perturbation theory techniques. However, using a semilocal functional in high-cation-disordered supercells may lead to the merging of valence bands and conduction bands. Single-shot hybrid calculations starting from metallic supercells can lead to inaccurate electronic structures.

### 3.6 Optical Spectra

**Figure 6:**
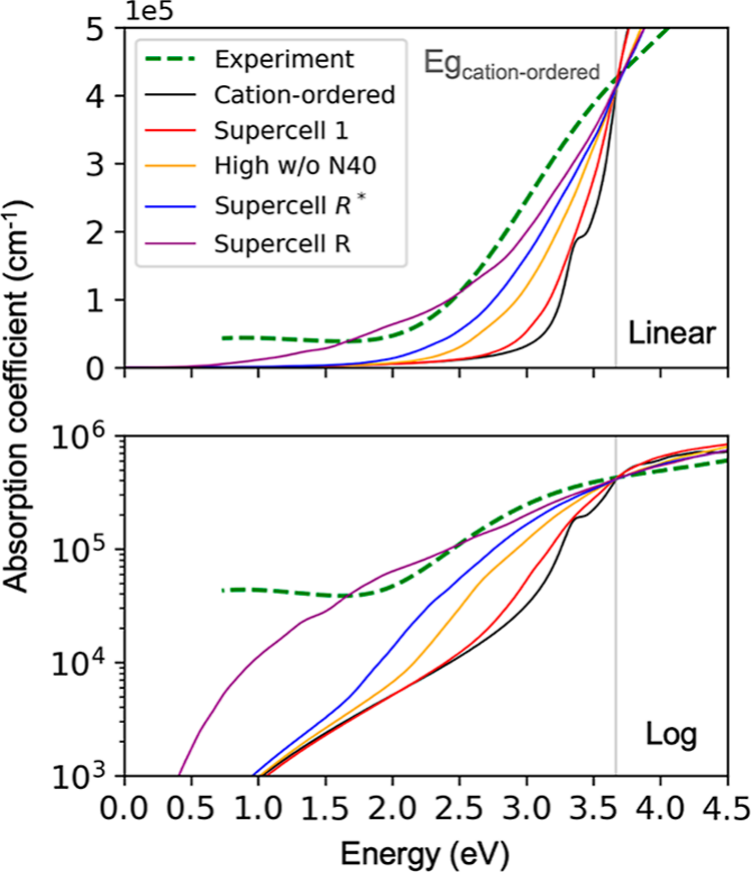
LR-TDDFT calculated and experimental absorption spectrum shown in linear (upper panel) and log (lower panel) *y*-axis. The experiment absorption onset is around 2 eV. The gray line represents the lowest direct band gap of cation-ordered ZnTiN_2_. The broad bump in the experimental curve from 0.5 to 2 eV is likely caused by free carrier absorption and other defect states arising from crystalline defects such as grain boundaries.

We used linear-response time-dependent DFT (LR-TDDFT) to compute the optical spectra of selected supercells. Figure 6 shows the calculated optical spectra with the use of the tuned global hybrid functional PBEα(0.147) along with a representative experimental optical absorption spectrum acquired by spectroscopic ellipsometry from a cation-disordered ZnTiN_2_ thin film. In cation-ordered ZnTiN_2_, we compute that the first exciton peak appears at 3.35 eV, 0.31 eV below the lowest direct band gap of 3.66 eV. In the low-cation-disordered supercell without N40 motifs (supercell 1), the calculated optical spectrum is close to the cation-ordered one without noticeable exciton peaks.

The absorption coefficient of Supercell *R* has the smallest onset value among our considered supercells, aligning with its lowest direct band gap of 0.60 eV, which largely originates from localized states associated with the N40 motifs. The optical spectra of the high-cation-disordered supercells without N40 [namely, Supercell R* and the high-cation-disordered supercell that includes 50% N22, 25% N13, and 25% N31 (denoted as High w/o N40 in Figure 6 with structure details provided in Supporting Information Figure S12)] show no peaks, more gradual slopes, and onsets somewhat larger than observed experimentally. These supercells (Supercells *R*, R*, and a high-cation-disordered one with 50% N22) have optical onsets within 0.5 eV, close to the experimental onset, showing an overall agreement.

Although none of the spectra of the cation-disordered supercells exhibit noticeable exciton peaks, there is a non-negligible spectral weight below the fundamental band edge, indicating the presence of bound excitons. Their first exciton states are located around 0.3 eV lower than their lowest direct gaps with three magnitudes of degrees lower oscillator strength than excitons in the cation-ordered crystal. The calculated optical spectra here neglect some important contributions, such as free carrier absorption, phonon-assisted absorption, and zero-point and temperature effects; however, these effects would tend to redshift the spectrum and increase absorption near the band gap, possibly leading to better agreement with the experiment.

## 4 Conclusions

In this work, we present a set of calculations and develop a physical picture of the origin of the band gap and electronic structure of recently synthesized cation-disordered semiconductor ZnTiN_2_. Using first-principles DFT calculations on 100 supercells, we demonstrate that cation disorder creates local charge imbalance near N and Ti atoms whose contribution dominates valence band and conduction band edges, respectively, leading to a broadening of the valence bands and conduction bands and reducing the band gap relative to cation-ordered ZnTiN_2_. N anions with a negatively charged environment shift the valence band edge upward, and Ti cations with a positively charged environment shift the conduction band edge downward. We investigate the energetics of various cation-disordered supercells and find that motifs where N is fully coordinated by Zn (N40) have high formation energies. These motifs also lead to localized states at valence band edges that could trap hole carriers. Our DFT–PBE valence band edge shifts are around 0.36 eV and conduction band edge shifts are around 0.34 eV in high-cation-disordered supercells without the N40 motifs. The states near conduction band edges are more localized in cation-disordered supercells, compared with the cation-ordered limit, suggesting lower electron carrier mobility. Our LR-TDDFT optical spectra also show good overall agreement with experiments for high-cation-disordered supercells. We hypothesize that the experimental systems should feature a high density of cation disorder, which is stabilized due to the limited kinetics and growth process. If the chemical environment of experimental systems exhibits minimal extreme inhomogeneities (like N40), the valence band and conduction band edge energies are expected to be similarly independent of structural arrangement, with fewer midgap states. Our work elucidates the impact that different degrees of cation disorder have on the electronic structure of ZnTiN_2_, which can be utilized in interpreting experimental findings, and our conclusions can be extended to interpret other heterovalent ternary nitrides, providing insights into optimal cation ordering manipulation.

## Supporting Information Available

Experimental synthesis and measurement details; lattice parameters of ZnTiN_2_ in theory and experiments; atomic structure of two cation-ordered ZnTiN_2_ phases; polarization of cation-ordered ZnTiN_2_; energy and band gap convergence with *k*-mesh densities and kinetic energy cutoffs in different functionals; band gap of cation-ordered ZnTiN_2_ calculated with different functionals; details for WOT-SRSH tuning; IPR of cation-ordered ZnTiN_2_; bond length, energetics, and electronic structure changes with the degree of cation disorder in cation-disordered ZnTiN_2_ supercells; charge density isosurface for a low- and a high-cation-disordered supercell; construction details and visualization for the atomic structure for selective cation-disordered ZnTiN_2_ supercells; and optical absorption convergence with *k*-mesh densities (PDF)
