# Supplementary material for: First-Principles Studies of the Electronic and Optical Properties of Zinc Titanium Nitride: The Role of Cation Disorder
Source: Chem Mater. 2024 Mar 25;36(7):3164–76. doi: 10.1021/acs.chemmater.3c02696 (PMC11008105; doi:10.1021/acs.chemmater.3c02696)
Supplement: Supplementary file 1 — cm3c02696_si_001.pdf [file cm3c02696_si_001.pdf]

Supporting Information for "First-principles studies of the electronic and optical properties of zinc titanium nitride: the role of cation disorder"

Sijia Ke,[*,†,‡] John S. Mangum,[¶] Andriy Zakutayev,[¶] Ann L. Greenaway,[¶] and Jeffrey B. Neaton[*,§,‖,⊥]

†*Department of Materials Science and Engineering, University of California at Berkeley, California 94720, USA*

‡*Chemical Sciences Division, Lawrence Berkeley National Laboratory, Berkeley, California 94720, USA*

¶*Materials, Chemistry, and Computational Science Directorate, National Renewable Energy Laboratory, Golden, Colorado 80401, USA*

§*Department of Physics, University of California at Berkeley, California 94720, USA*

‖*Materials Sciences Division, Lawrence Berkeley National Laboratory, Berkeley, California 94720, USA*

⊥*Kavli Energy NanoSciences Institute at Berkeley, Berkeley, California 94720, USA*

E-mail: sijia_ke@berkeley.edu; jbneaton@lbl.gov

Experimental synthesis and measurement

Thin films of Zn-Ti-N were deposited by radio frequency co-sputtering in a custom vacuum chamber containing a cryoshroud surrounding the plasma zone. Films were deposited for 2 hours at ambient temperature onto an EXG glass substrate using Zn and alloy ZnTi 2"

diameter targets. RF power densities on each target were tuned such that samples containing nominally stoichiometric films with one-to-one Zn:Ti ratios were produced. A chamber base pressure $<4\times10^{-7}$ Torr was reached prior to flowing 20 sccm of Ar and 10 sccm of N_2 while maintaining a chamber pressure of 3.5×10^{-3} Torr by controlling a partially closed gate valve to the turbomolecular pump.

Spectroscopic ellipsometry data were acquired using a J.A. Woollam Co. M-2000 variable angle ellipsometer at incident angles of 65°, 70°, and 75° over a photon energy range of 0.73–6.46 eV. Raw Φ and Δ data were modeled and fit using the CompleteEASE software (version 6.63) to extract optical parameters n, k, and absorption coefficient. A generalized oscillator model was constructed using a PSemi-M0 oscillator to fit the $ZnTiN_2$ optical absorption edge and a single Drude oscillator to fit the sub-gap free carrier absorption region below 2 eV.

Cation-ordered $ZnTiN_2$ atomic and electronic structure

Table S1: Lattice parameters (in Å) of $ZnTiN_2$ calculated with DFT-PBE and measured from experiments.[1] The experimental lattice parameters measured for wurtzite $ZnTiN_2$ are a = 3.1 Å, c = 5.0 Å, and converting to the orthorhombic setting gives values in the table.

Lattice	Theory (ordered; VASP)	Theory (ordered; QE)	Experiment (disordered)
a	5.71	5.70	5.4
b	6.59	6.57	6.2
c	5.26	5.23	5.0

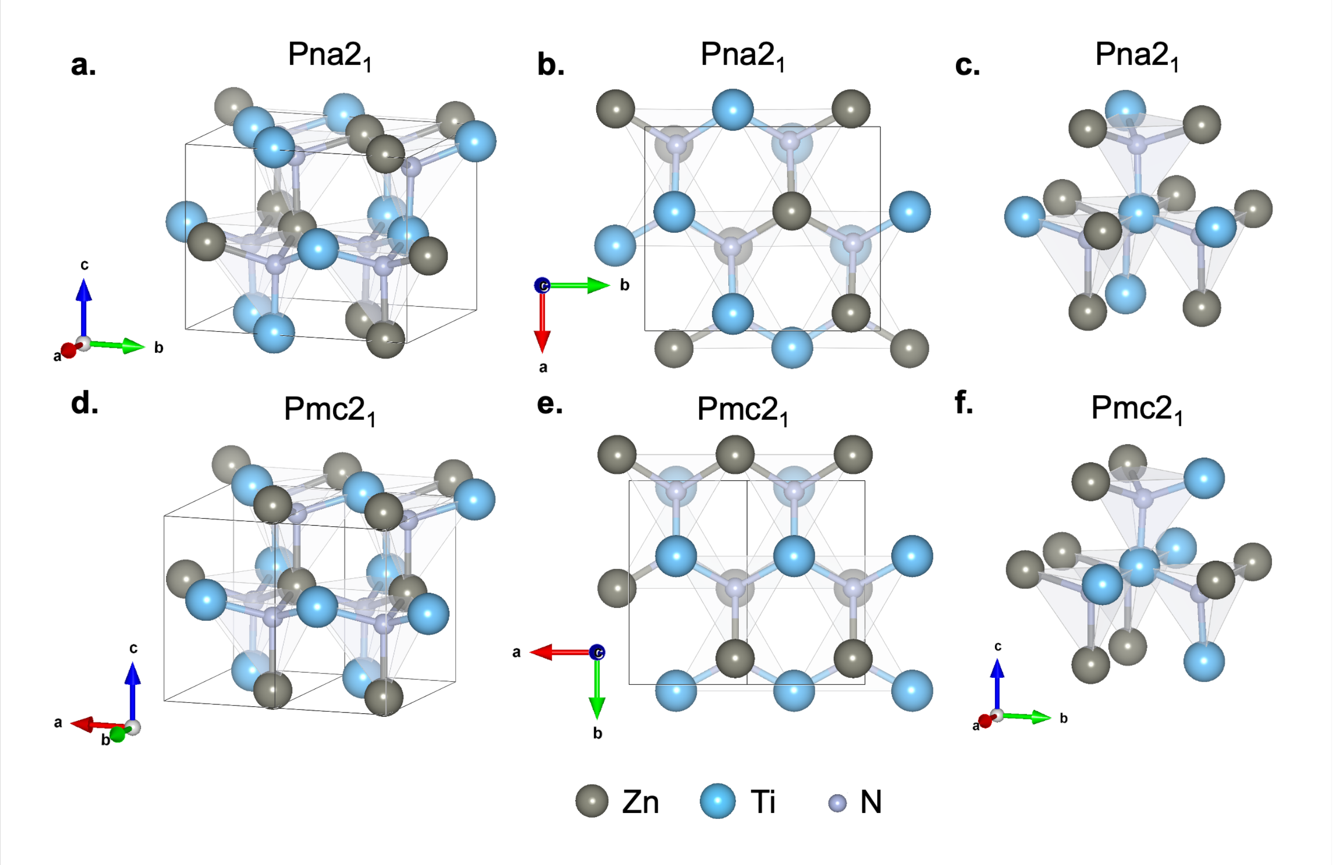

Figure S1: Atomic structures of $Pna2_1$ (a, b, c) and $Pmc2_1$ (d, e, f), the latter of which is 16.4 meV/f.u. higher in energy than the former. The $Pmc2_1$ structure only contains 2 f.u. so we display two primitive cell in d, e for better comparison.

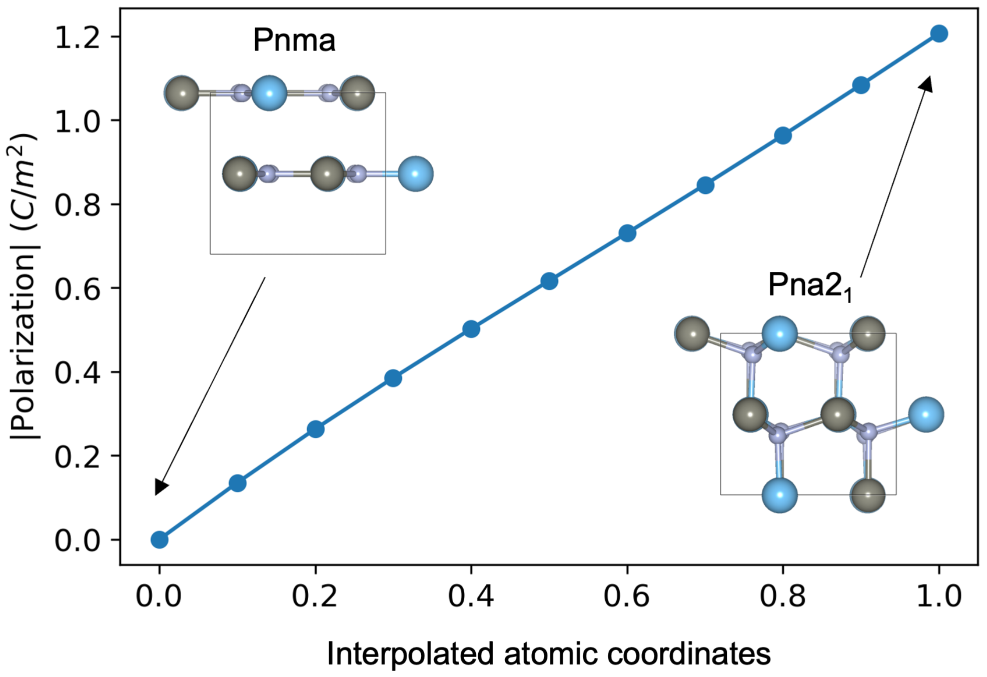

Figure S2: Polarization versus interpolated atomic coordinates between the hexagonal reference structure *Pnma* and the ground structure of cation-ordered $ZnTiN_2$, $Pna2_1$.

Energy and band gap convergence

Table S2: Energy and band gap convergence with k-mesh density in VASP. Values are obtained in primitive cation-ordered $ZnTiN_2$ using PBE functional

K-mesh	Energy per atom /eV	Band gap / eV
$4 \times 5 \times 5$	-7.1829991	2.259
$6 \times 8 \times 8$	-7.1829941	2.267
$8 \times 10 \times 10$	-7.1829955	2.254
$10 \times 12 \times 12$	-7.1829957	2.248

Table S3: Energy and band gap convergence with k-mesh density in cation-disordered $ZnTiN_2$ supercell R using HSE06 functional in VASP. The energy reference is the energy value with$4 \times 4 \times 4$.

K-mesh	Relative Energy per atom /eV	Band gap / eV
$1 \times 1 \times 1$	-0.003491	0.515
$2 \times 2 \times 2$	0.000163	0.448
$4 \times 4 \times 4$	0.000000	0.448

Table S4: Energy difference and band gap convergence with kinetic energy cutoff in VASP. Energy and band gap values are obtained self-consistently in primitive cation-ordered $ZnTiN_2$ using PBE functional. The energy difference is created by displacing an 0.1 Å atomic distortion of one Zn in cation-ordered $ZnTiN_2$.

Kinetic energy cutoff / eV	Energy difference per atom /eV	Band gap / eV
400	0.0032805	2.253
500	0.0032847	2.254
600	0.0032798	2.254

Electronic structure calculated from different functionals

Table S5: Band gap calculated in self-consistent calculations with different functionals using both VASP and QE. The second and third colomn band gap calculations do not use the same structure. Two cation-ordered structures are individually relaxed with PBE in each code and are used to obtain band gaps, the lattice parameters of which are tabulated in Table S1.

Functional	Band gap (VASP)/ eV	Band gap (QE)/ eV	K mesh
LDA	2.20	2.38	8×10×10
PBE	2.25	2.41	8×10×10
PBEα(0.147)	3.31	3.49	4×5×5
HSE06	3.37	3.57	4×5×5
WOT-SRSH	3.41	3.52	4×5×5
PBE0	4.08	4.27	4×5×5

WOT-SRSH tuning

We utilize the Wannier-localized optimally-tuned screened range-separated hybrid (WOT-SRSH) functional[2–4] to obtain an accurate fundamental band gap for cation-ordered $ZnTiN_2$. WOT-SRSH is nonempirical and has the correct long-range Coulomb screening and satisfies an ionization potential (IP) ansatz.[5] In a range-separated hybrid functional, the exchange part of Coulomb potential is written

$$\frac{1}{r} = \frac{\alpha + \beta \mathrm{erf}(\gamma r)}{r} + \frac{1 - [\alpha + \beta \mathrm{erf}(\gamma r)]}{r}, \quad (1)$$

where the first term is calculated with Fock exchange and the second term is treated with an appropriate semi-local Kohn-Sham exchange. There are three parameters, α, β, and γ. $\alpha + \beta = \frac{1}{\epsilon_\infty}$ is set to obtain the correct long-range Coulomb screening. Therefore, only two parameters, α and γ are left to be determined. α is set to 0.25, and γ is set by fulfilling IP ansatz, which is equivalent to $\Delta I = 0$ from the orange curve in Figure S3, where ΔI means the energy difference between the energy of removed function and ionization energy for removing such function. There can be multiple $\{\alpha,\gamma\}$ pairs that give $\Delta I = 0$. We use $\alpha = 0.25$, $\beta = -0.103$ and $\gamma = 1.07$ bohr^{-1} as the WOT-SRSH parameters in Table S5. For the details of the WOT-SRSH procedure, we reference prior work.[2]

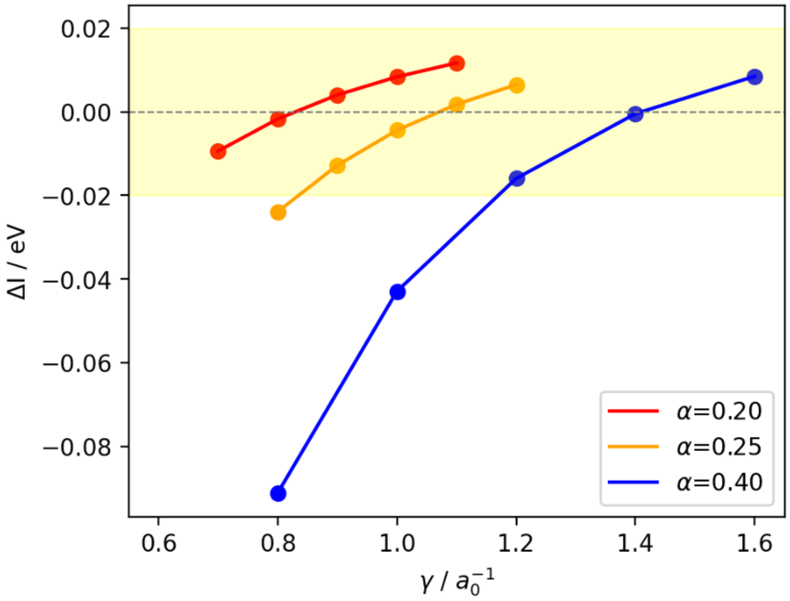

Figure S3: ΔI as a function of range separation parameter γ at the fraction of exact exchange in short-range $\alpha = 0.20, 0.25, 0.40$ in cation-ordered $ZnTiN_2$. When $\Delta I = 0$, the IP ansatz is satisfied.

Inverse participation ratio

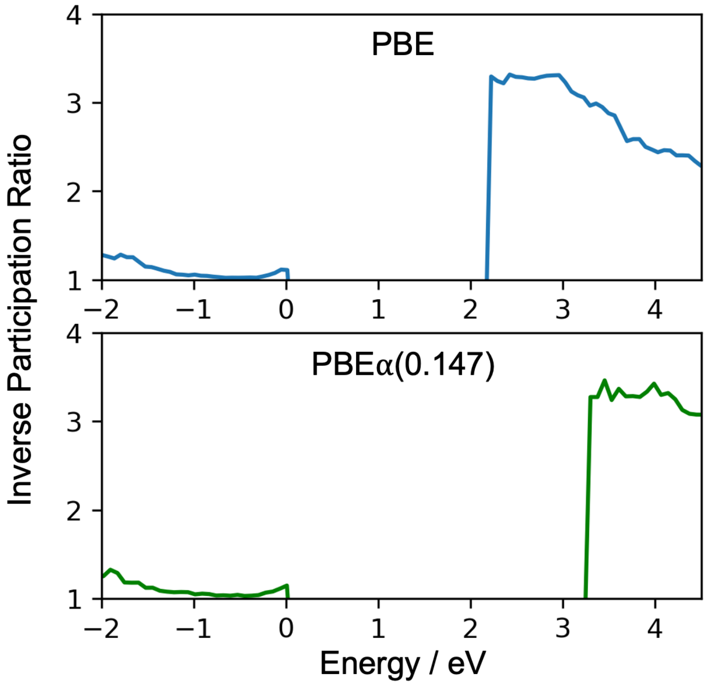

Figure S4: Inverse participation ratio near band edges calculated from PBE and PBE$\alpha(0.147)$ in cation-ordered $ZnTiN_2$. The similarity of IPR values calculated from different functionals suggests the sufficiency of using PBE to study IPR changes.

Bond length, energetics, electronic structure changes in cation-disordered $ZnTiN_2$ supercells

Here we comment on the choice of fixed lattice parameters. We relax the lattice parameters of Supercell R^* since it contains the highest cation disorder density among all considered supercells except for Supercell R. Compared to the unrelaxed lattice, the a and b lattice parameters both increase by 0.7%, while the c lattice parameter decreases by 3%. The change of lattice parameters brings the structure closer to the wurtzite structure. The relaxed total energy is 1.8 meV per f.u. lower and the band gap is 0.06 eV lower than the unrelaxed one. The changes due to lattice relaxation are small enough that our choice of fixed lattice parameters is reasonable.

In Figure S5, three cation-disordered supercells are plotted. The distribution of N-Zn and N-Ti bond length broadens with a higher degree of cation disorder. As shown in Figure

S5 c, N-Zn and N-Ti bond lengths are generally larger if N is the center of a more positively charged motif. Such bond length change counteracts the energetically unfavorable chemical environment.

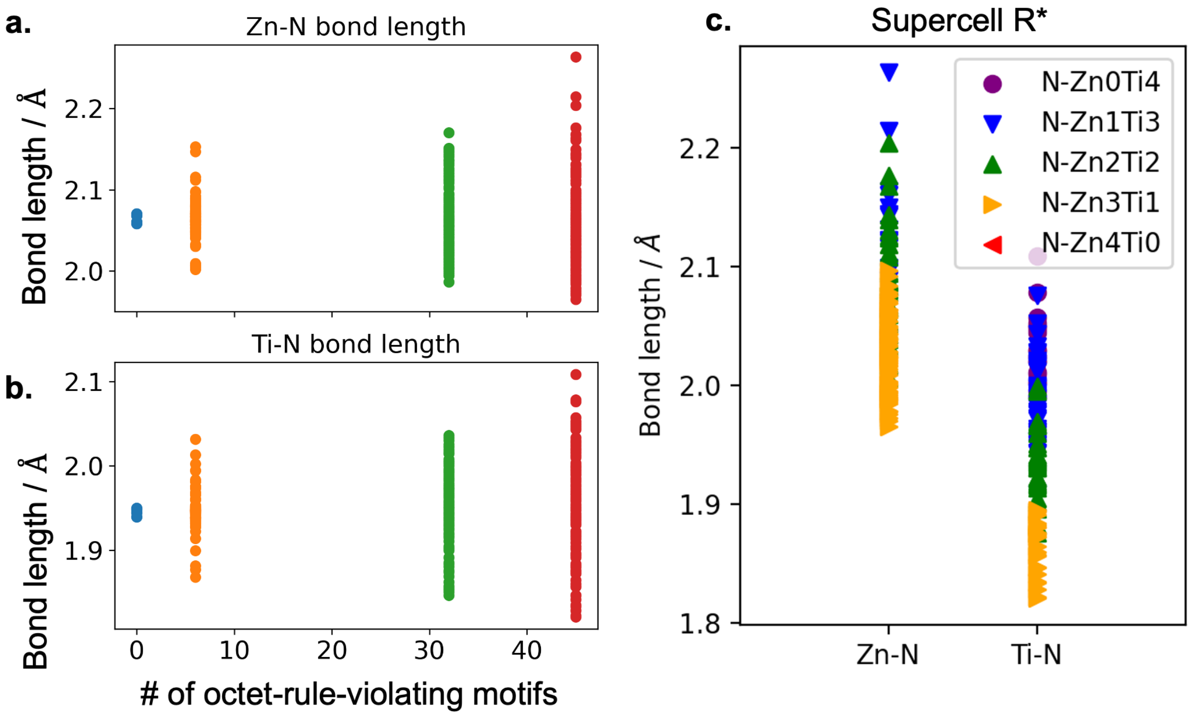

Figure S5: Bond length distribution in cation-disordered supercells. a-b. The Zn-N and Ti-N bond length distribution from cation-ordered crystal and 3 other selected supercells, Supercell 1, R^*, and a high cation-disordered one (25% N31, 50% N22, 25% N13). Each color represents a different supercell. c. Zn-N and Ti-N bond length distributions from Supercell R^* (0 N40, $\frac{25}{64}$ N31, $\frac{19}{64}$ N22, $\frac{15}{64}$ N13, $\frac{5}{64}$ N04) (which are also shown as the red circles in a-b). The marker indicates the Zn-N or Ti-N belonged N motif type. Zn-N and Ti-N bond lengths decrease (increase) when N is the centered N of a less (more) positively charged motif.

Table S6: Energy and band gap of several low cation-disordered $ZnTiN_2$ supercells.

No.	N40	N31	N22	N13	N04	ΔTi-Ti	Energy/f.u. (meV)	E_g^{PBE} (eV)	VBM shift(eV)	CBM shift(eV)	$E_g^{PBE\alpha(0.147)}$ (eV)
13	1	3	55	5	0	0.5	63.0	1.276	0.741	0.232	2.373
14	1	3	55	5	0	0.5	58.3	1.365	0.780	0.104	2.471
15	1	3	55	5	0	0.5	57.1	1.457	0.665	0.128	2.530
16	1	3	55	5	0	0.5	56.7	1.364	0.661	0.226	2.435
17	0	5	55	3	1	0.5625	45.4	1.853	0.182	0.216	2.931
18	0	5	55	3	1	0.5625	44.7	1.843	0.151	0.257	2.946

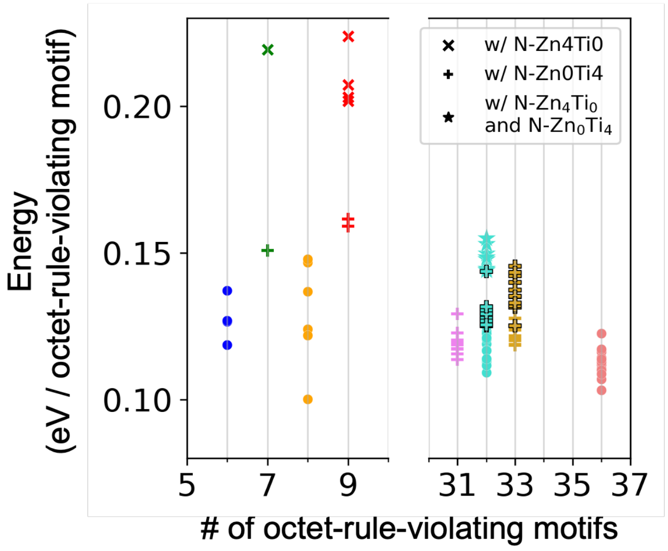

Figure S6: Relative energy of cation-disordered $ZnTiN_2$ compared to cation-ordered crystal per octet-rule-violating motif. Except for supercells with N40, the relative energies are similar among low and high cation-disordered supercells.

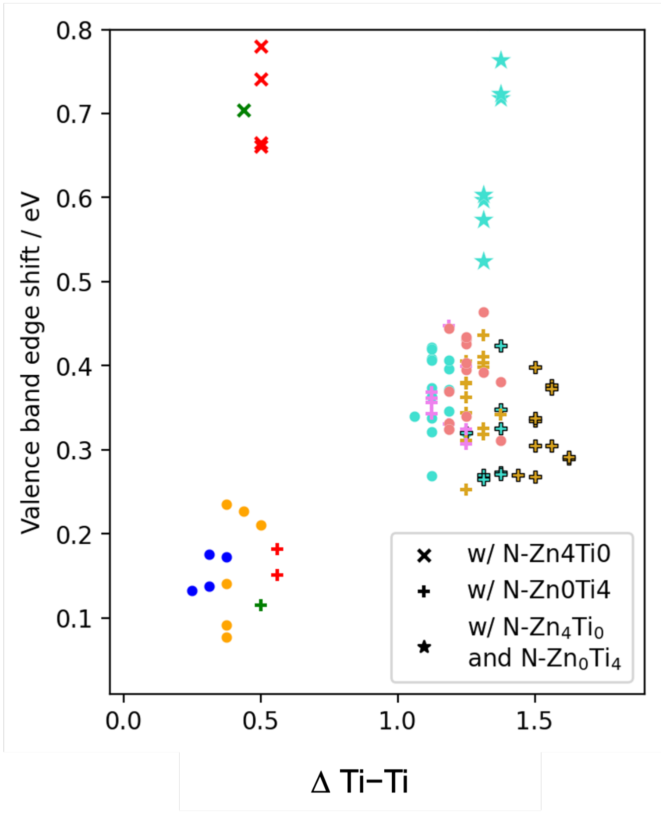

Figure S7: Relative valence band edge shift calculated with PBE versus ΔTi-Ti (defined in main text Eq. 1). Unlike the conduction band edge, the absolute shift of the valence band edge does not show a clear general trend with ΔTi-Ti.

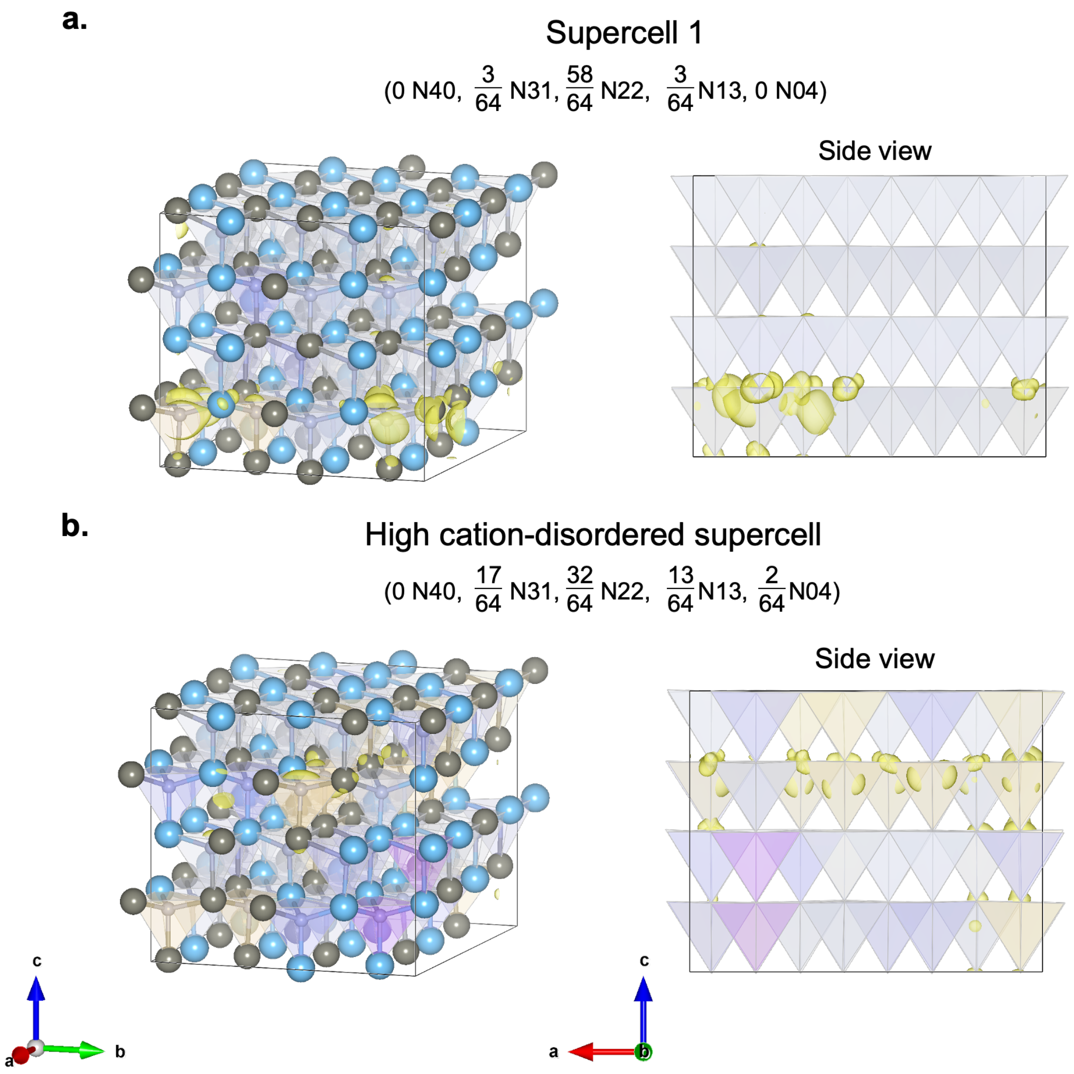

Figure S8: Charge density isosurface of valence band maximum at Supercell 1 (a) and high cation-disordered supercell (b) whose density of states is plotted in Figure 5 d. The charge density of high cation-disordered is more widespread than Supercell 1. The yellow tetrahedral color means N31 motif, dark blue means N13, and purple means N04.

Atomic structure of selected cation-disordered $ZnTiN_2$ supercells

As the motivation behind the construction of various cation-disordered supercells is mentioned in the main text, we discuss some details of our supercell construction here. A close swap shown here refers to the creation of antisite defects between two neighboring cations. The 18 supercells in the low cation disorder range (only 6-9 octet-rule-violating motifs per supercell) include most of the possible atomic configurations in that range for 128-atom supercell sizes. Starting with 1 close Zn and Ti swap, Supercells $1-4$ are all four possible atomic configurations (see SI Figure S9) containing 3 ($\frac{3}{64} \approx 4.69\%$) N13 and 3 N31 motifs, with slight atomic structure differences. Based on these four possible structures, an additional close swap can create two supercells with 57 ($\frac{57}{64} \approx 89.06\%$) N22, one with 1 ($\frac{1}{64} \approx 1.56\%$) N40 (Supercell 5), and one with 1 N04 (Supercell 6). We also construct 6 supercells containing 56 ($\frac{56}{64} = 87.5\%$) N22 (Supercells $7-12$), which are all possible configurations with 56 N22 created by adding one close swap on Supercell 1. In addition, another 6 supercells with 55 ($\frac{55}{64} \approx 86.94\%$) N22 and N04 (or N40) are constructed, which information is in SI Table S2. The detailed atomic structures are shown in SI Figure S9-S11.

We construct high cation-disordered supercells by applying one or two random close swaps to a specific cation-disordered supercell with 32 ($\frac{32}{64} = 50\%$) charge-neutral N22 motifs. These supercells contain 28-33 N22, 0-3 N04, and 0-1 N40 motifs. It is impossible and unnecessary to examine every high cation-disordered configuration. One close swap influences the local charge balance of 6 motifs, therefore the structure difference created by two random close swaps in 128-atom high cation-disordered supercells can sample the possible atomic configuration space. Here we note that the cation disorder of the experimental systems might not be located in the considered density range. However, the high cation-disordered supercells decouple positive and negative charge unbalanced motifs. The statistical probability of the number of octet-rule-violating motifs created by performing an additional close swap is close

to zero at high cation disorder (and high temperatures), unlike the low cation disorder range we consider. We also use quasi-random numbers to determine cation occupation to create one randomly distributed cation-disordered supercell (Supercell R). Another supercell (Supercell R^*) is created by applying extra quasi-random Zn-Ti swaps to remove existing N40 and increase the octet-rule-obeying N22 motif density inside the random supercell (Supercell R).

Table S7: High cation-disordered $ZnTiN_2$ supercell structure details.

N40	N31	N22	N13	N04	ΔTi-Ti	# of supercells
0	18	28	18	0	1.1875-1.375	15
0	17	31	15	1	1.25-1.375	14
0	18	31	12	3	1.4375-1.625	11
0	16	32	16	0	1.0625-1.25	15
0	17	32	13	2	1.25-1.375	8
1	15	32	15	1	1.3125-1.375	8
0	16	33	14	1	1.125-1.25	9

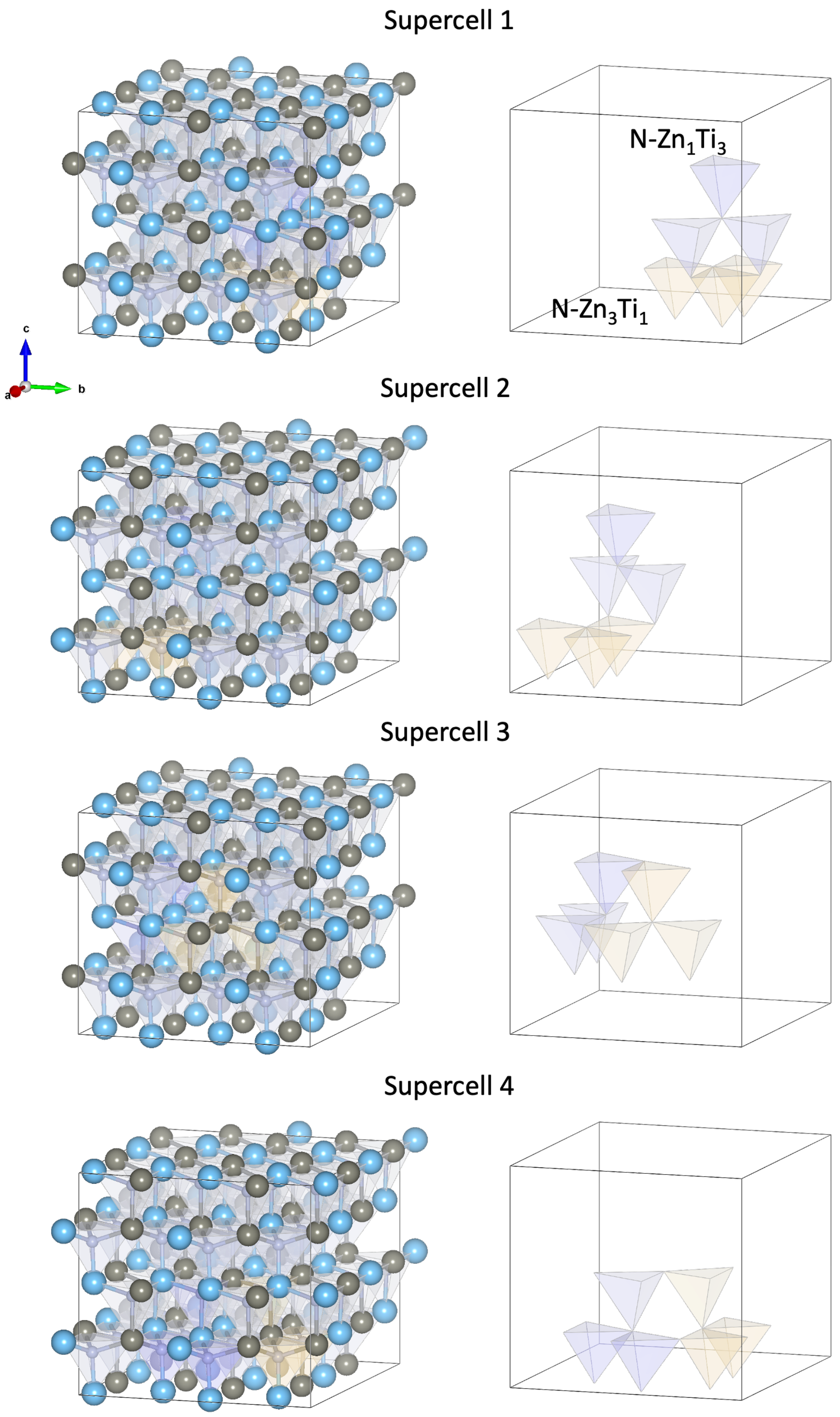

Figure S9: Atomic structures of Supercell 1-4. In the right panel, only octet-rule-violating motifs are shown in color. Yellow means N31 and dark blue means N13.

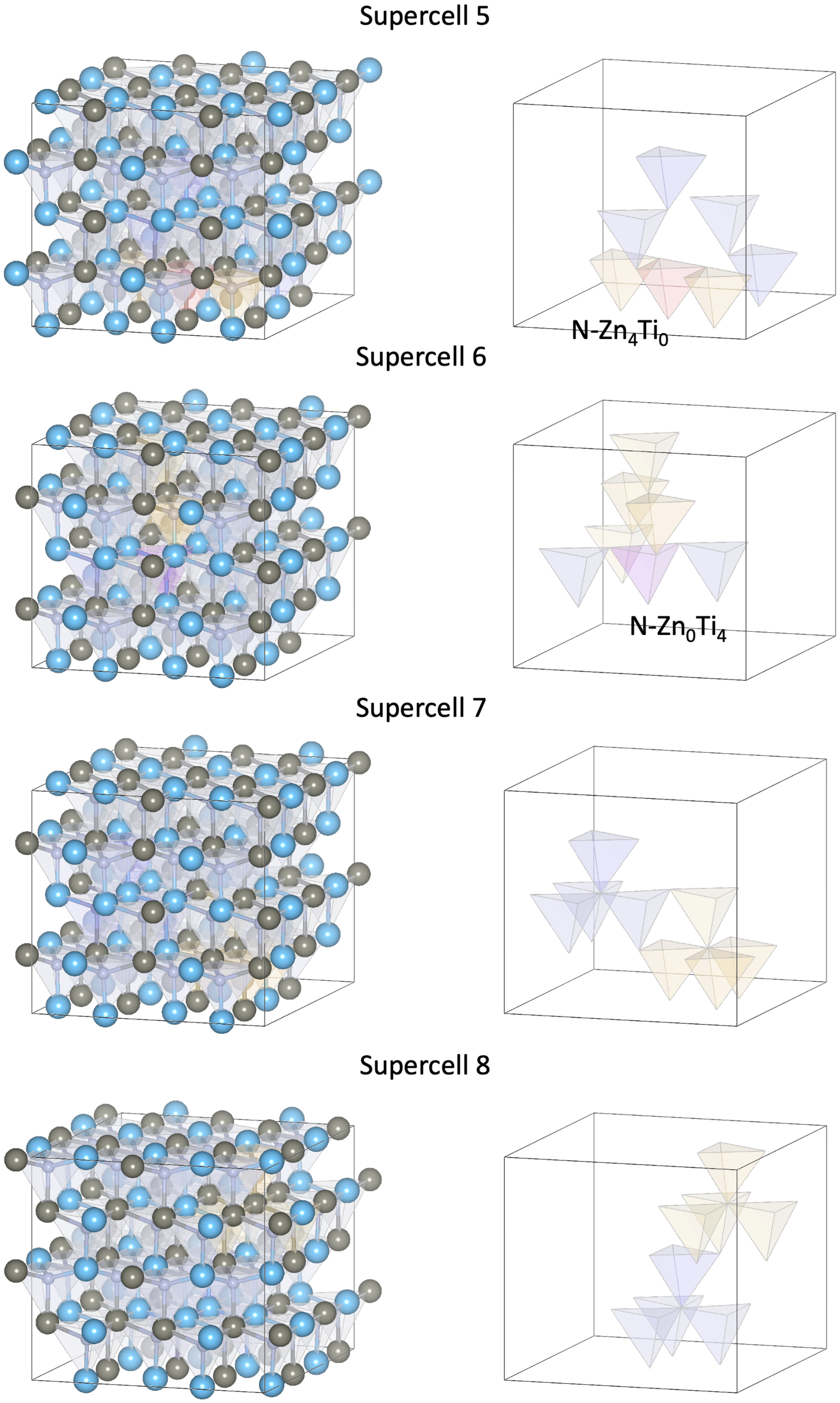

Figure S10: Atomic structures of Supercell 5-8. In the right panel, only octet-rule-violating motifs are shown in color. Red means N40, yellow means N31, dark blue means N13, and purple means N04.

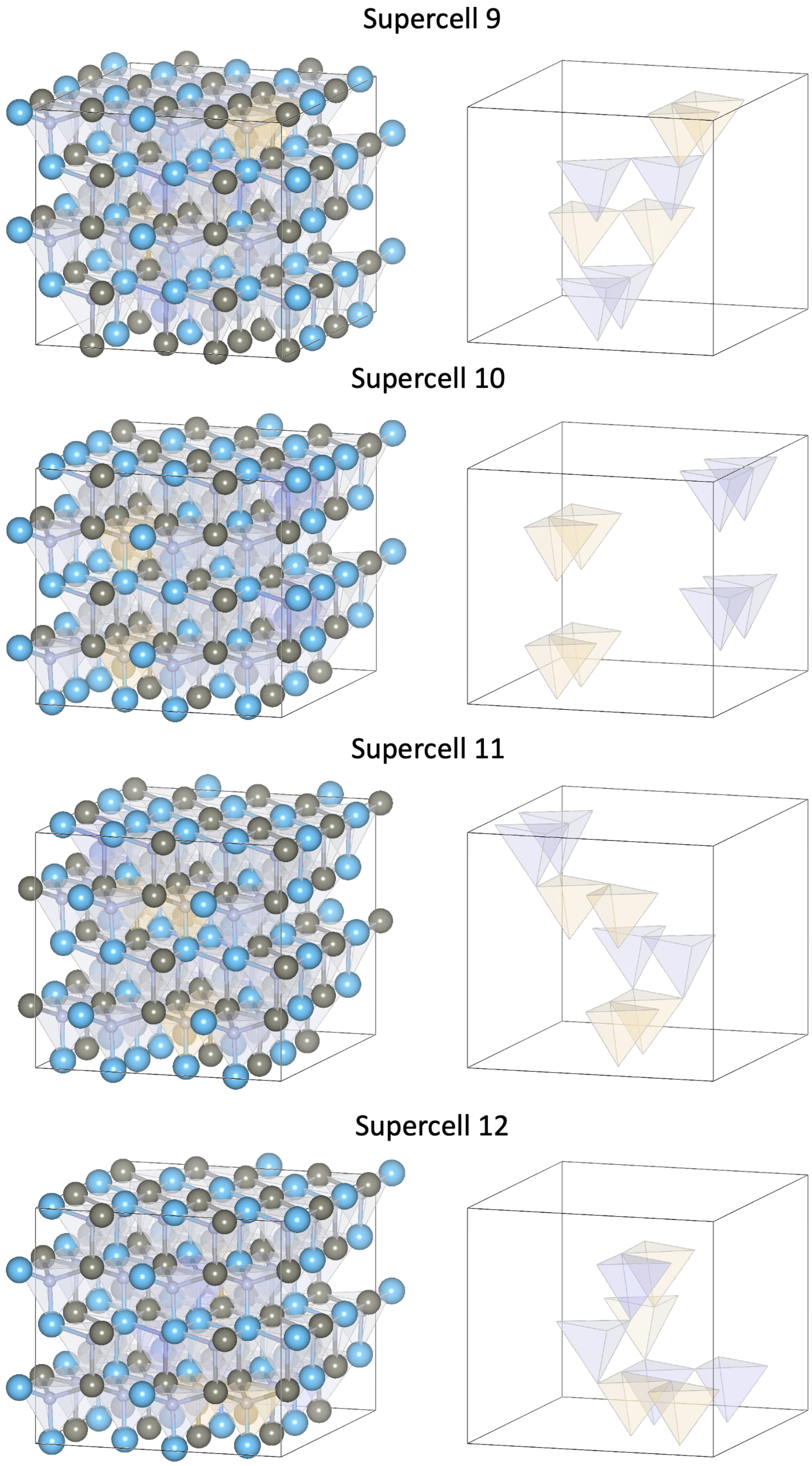

Figure S11: Atomic structures of Supercell 9-12. In the right panel, only octet-rule-violating motifs are shown in color. Red means N40, yellow means N31, dark blue means N13, and purple means N04.

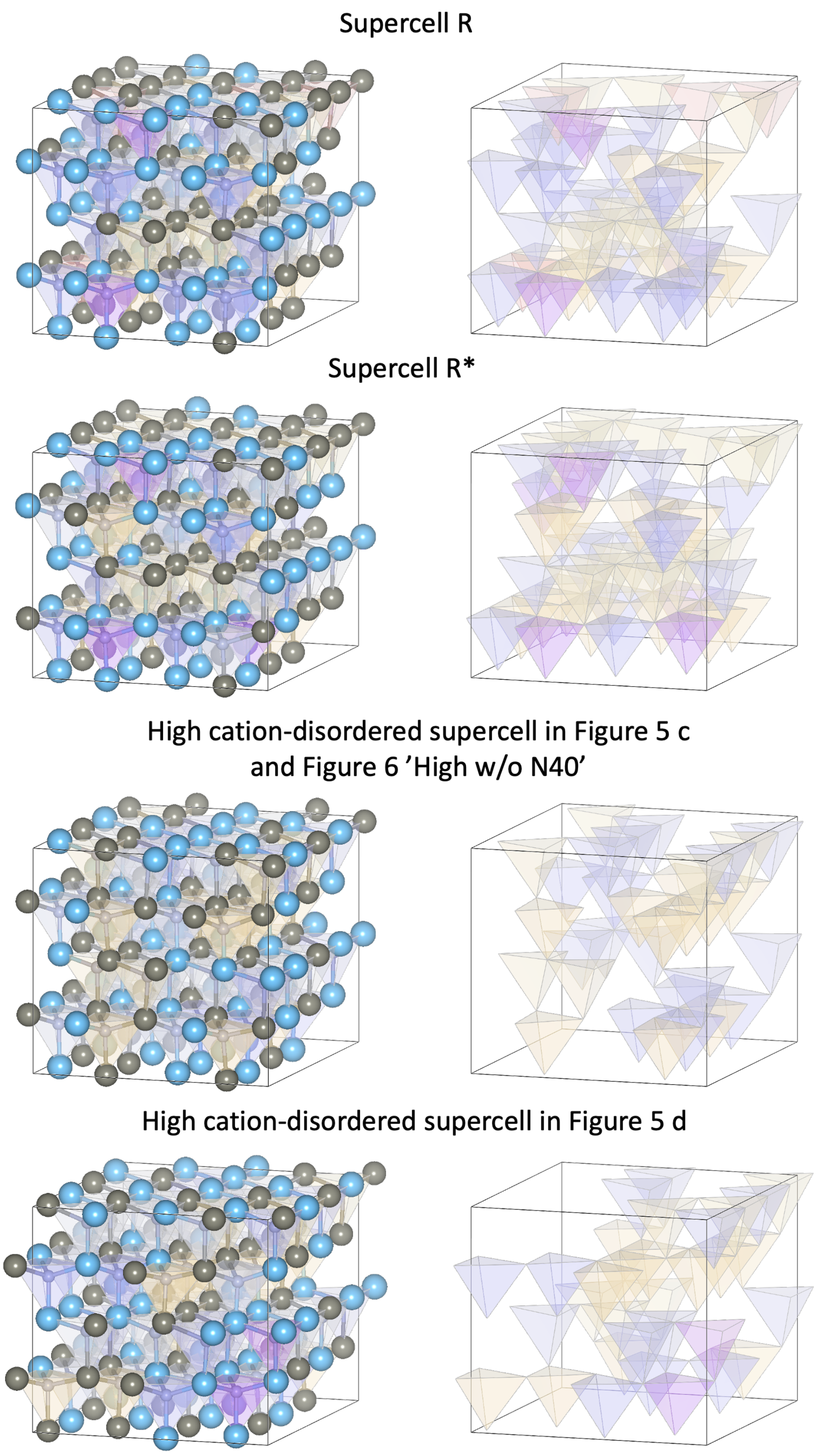

Figure S12: Atomic structures of Supercell R, R^*, and two high cation-disordered supercells shown in Figure 5 c d and Figure 6. In the right panel, only octet-rule-violating motifs are shown in color. Red means N40, yellow means N31, dark blue means N13, and purple means N04.

Optical absorption convergence

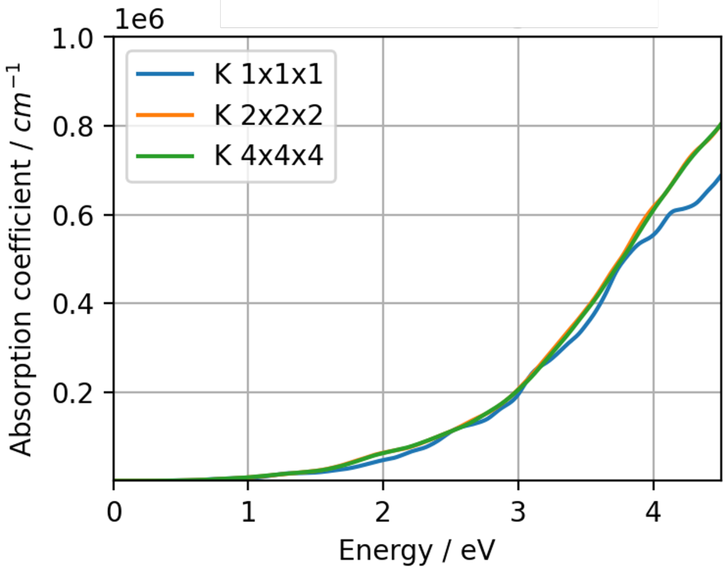

Figure S13: The LR-TDDFT optical absorption spectra along z direction of a 128-atom cation-disordered supercell convergence with K mesh density.

We average the absorption spectra over three orientations since the experimental sample is polycrystalline. The absorption coefficient is calculated from the imaginary and real dielectric functions in LR-TDDFT results. Small off-diagonal components of dielectric function exist in some energy range but are less than 10% of the diagonal components. We ignore the contribution of these small off-diagonal components in computing the absorption coefficients in Figure 6.

References

(1) Greenaway, A. L. et al. Zinc Titanium Nitride Semiconductor toward Durable Photoelectrochemical Applications. *Journal of the American Chemical Society* **2022**, *144*, 13673–13687.

(2) Wing, D.; Ohad, G.; Haber, J. B.; Filip, M. R.; Gant, S. E.; Neaton, J. B.; Kronik, L.

Band Gaps of Crystalline Solids from Wannier-localization–Based Optimal Tuning of a Screened Range-Separated Hybrid Functional. *Proceedings of the National Academy of Sciences* **2021**, *118*, e2104556118.

(3) Gant, S. E.; Haber, J. B.; Filip, M. R.; Sagredo, F.; Wing, D.; Ohad, G.; Kronik, L.; Neaton, J. B. Optimally Tuned Starting Point for Single-Shot *GW* Calculations of Solids. *Physical Review Materials* **2022**, *6*, 053802.

(4) Ohad, G.; Wing, D.; Gant, S. E.; Cohen, A. V.; Haber, J. B.; Sagredo, F.; Filip, M. R.; Neaton, J. B.; Kronik, L. Band Gaps of Halide Perovskites from a Wannier-localized Optimally Tuned Screened Range-Separated Hybrid Functional. *Physical Review Materials* **2022**, *6*, 104606.

(5) Ma, J.; Wang, L.-W. Using Wannier Functions to Improve Solid Band Gap Predictions in Density Functional Theory. *Scientific Reports* **2016**, *6*, 24924.
